# Antiviral activity of the host defense peptide piscidin 1: investigating a membrane-mediated mode of action

**DOI:** 10.3389/fchem.2024.1379192

**Published:** 2024-06-26

**Authors:** Tristan Bepler, Michael D. Barrera, Mary T. Rooney, Yawei Xiong, Huihui Kuang, Evan Goodell, Matthew J. Goodwin, Elizabeth Harbron, Riqiang Fu, Mihaela Mihailescu, Aarthi Narayanan, Myriam L. Cotten

**Affiliations:** ^1^ New York Structural Biology Center, New York, NY, United States; ^2^ School of Systems Biology, George Mason University, Manassas, VA, United States; ^3^ Department of Applied Science, William & Mary, Williamsburg, VA, United States; ^4^ Department of Chemistry, Hofstra University, Hempstead, NY, United States; ^5^ Department of Chemistry, William & Mary, Williamsburg, VA, United States; ^6^ National High Magnetic Field Laboratory, Tallahassee, FL, United States; ^7^ Institute for Bioscience and Biotechnology Research, Rockville, MD, United States; ^8^ Department of Biology, George Mason University, Manassas, VA, United States; ^9^ Department of Biochemistry and Biophysics, Oregon State University, Corvallis, OR, United States

**Keywords:** antiviral host defense peptide, cholesterol, liquid ordered and disordered phases, lipid mixing, membrane disruption, membrane heterogeneity, membrane thinning, viral envelopes

## Abstract

Outbreaks of viral diseases are on the rise, fueling the search for antiviral therapeutics that act on a broad range of viruses while remaining safe to human host cells. In this research, we leverage the finding that the plasma membranes of host cells and the lipid bilayers surrounding enveloped viruses differ in lipid composition. We feature Piscidin 1 (P1), a cationic host defense peptide (HDP) that has antimicrobial effects and membrane activity associated with its N-terminal region where a cluster of aromatic residues and copper-binding motif reside. While few HDPs have demonstrated antiviral activity, P1 acts in the micromolar range against several enveloped viruses that vary in envelope lipid composition. Notably, it inhibits HIV-1, a virus that has an envelope enriched in cholesterol, a lipid associated with higher membrane order and stability. Here, we first document through plaque assays that P1 boasts strong activity against SARS-CoV-2, which has an envelope low in cholesterol. Second, we extend previous studies done with homogeneous bilayers and devise cholesterol-containing zwitterionic membranes that contain the liquid disordered (L_d_; low in cholesterol) and ordered (L_o_, rich in cholesterol) phases. Using dye leakage assays and cryo-electron microscopy on vesicles, we show that P1 has dramatic permeabilizing capability on the L_o_/L_d_, an effect matched by a strong ability to aggregate, fuse, and thin the membranes. Differential scanning calorimetry and NMR experiments demonstrate that P1 mixes the lipid content of vesicles and alters the stability of the L_o_. Structural studies by NMR indicate that P1 interacts with the L_o_/L_d_ by folding into an α-helix that lies parallel to the membrane surface. Altogether, these results show that P1 is more disruptive to phase-separated than homogenous cholesterol-containing bilayers, suggesting an ability to target domain boundaries. Overall, this multi-faceted research highlights how a peptide that interacts strongly with membranes through an aromatic-rich N-terminal motif disrupt viral envelope mimics. This represents an important step towards the development of novel peptides with broad-spectrum antiviral activity.

## Introduction

The Coronavirus disease 2019 (COVID-19) pandemic has had a devastating impact worldwide. According to the World Health Organization, the death toll exceeded 6.9 million at the end of 2023. While vaccination against the severe acute respiratory syndrome coronavirus 2 (SARS-CoV-2) that causes COVID-19 has saved tens of millions of lives globally, concerns remain that even mild infections, including breakthrough infections in vaccinated subjects, may lead to lingering health issues (Amanatidou et al., 2022; Lipsitch et al., 2022; Rahman et al., 2022; Zheng et al., 2022; Davis et al., 2023; Perumal et al., 2023). It is also the case that a large portion of the world population is not vaccinated against SARS-CoV-2 and emerging viruses constitute a growing threat to human health (Sallam, 2021; Rahman et al., 2022; Lazarus et al., 2023). This context is fueling the search for broad-spectrum antiviral therapeutics that could complement existing vaccines in the fight against emerging and reemerging viruses (Harrison, 2008; Porotto et al., 2010; Wolf et al., 2010; Miller et al., 2011; Schoggins and Randall, 2013; Vigant et al., 2013; Hollmann et al., 2014; Vigant et al., 2015; Zhao et al., 2016; Park and Gallagher, 2017; Wang et al., 2018; Momattin et al., 2019; Wiehe et al., 2019; Baglivo et al., 2020; Chowdhury et al., 2020; Li and De Clercq, 2020; Schütz et al., 2020; Zeng et al., 2020; Saud et al., 2022). In this research, we focus on enveloped viruses and explore the ability of membrane-active agents, such as motif-containing host defense peptides (HDPs), to disrupt their envelopes.

Enveloped viruses dangerous to humans include SARS-CoV-2 as well as Human Immunodeficiency Virus Type 1 (HIV-1). These viruses infect cells by fusing their lipid envelope with the plasma or endosomal membrane of the host (Lorizate and Kräusslich, 2011; Plemper, 2011; Schoggins and Randall, 2013). While interactions between host and viral proteins play a major role in viral infection, other key players include the lipid envelope that surrounds virions (Pollock et al., 2010; Wolf et al., 2010; Martín-Acebes et al., 2013; Miguel et al., 2013; Schoggins and Randall, 2013; Vigant et al., 2015; Yang et al., 2016; Yan et al., 2019). Viruses acquire lipids for their envelopes in a way that depends on the assembly and egress pathways involving host components (Brügger et al., 2006; Jolly and Sattentau, 2007; Heaton and Randall, 2011; Lorizate and Kräusslich, 2011; Hogue et al., 2014; Alsaadi and Jones, 2019; Ghosh et al., 2020; Saud et al., 2022). With great strides made recently in lipidomics, a growing number of viral lipodomes have become available, shedding light on the complex chemical composition of viral envelopes (Chan et al., 2010; Heaton and Randall, 2011; Ivanova et al., 2015; Kyle, 2021). In particular, viral envelopes and plasma membranes differ in chemical content, and therefore physicochemical properties (e.g., curvature; fluidity). Notably, the envelope of HIV-1 is enriched in cholesterol (Chol), sphingomyelin (SM), and phosphatidylserine (PS) compared to the plasma membrane from which it buds (Brügger et al., 2006; Jolly and Sattentau, 2007). With a high Chol content, the HIV-1 envelope is expected to contain ordered microdomains, also called lipid rafts. In contrast to HIV-1, SARS-CoV-2 follows a lysosomal egress pathway after leaving the endoplasmic reticulum (ER)/Golgi intermediate complex (Ghosh et al., 2020). A recent study suggests that its envelope features lower Chol and higher phosphatidylinositol (PI) content than the ER and Golgi (Saud et al., 2022). For instance, SARS-CoV-2 derived from *Vero* and A549 cells contains 18% and 37% PI, respectively, while the plasma membrane content is typically below 10%. In these two respective cell lines, the Chol/phospholipid ratios of the virus are 0.0006 and 0.005 while this ratio is 0.76 in the plasma membrane. Another differentiating factor when considering host and viral membranes is that the biogenic reparative ability associated with metabolically active cells is lacking in viruses (Hanson et al., 2016; Zeng et al., 2020; Saud et al., 2022). Overall, the differentiating factors between the membranes of enveloped viruses and host cell organelles provide fertile ground to design agents that specifically target enveloped viruses over host cells. Recently, several studies have indicated that broad-spectrum antiviral action can be achieved through membrane-active compounds that disrupt viral envelopes (Wolf et al., 2010; Vigant et al., 2013; Saud et al., 2022).

In this study, we explore a peptide-based approach to disrupt enveloped viruses such as SARS-CoV-2 and HIV-1. For this purpose, the focus is on Piscidin 1 (P1, FFHHIFRGIVHVGKTIHRLVTG), an amphipathic HDP derived from fish (Silphaduang and Noga, 2001; Lauth et al., 2002). Most membrane-active HDPs, including P1, are cationic, enabling them to preferentially associate with the anionic membranes of bacteria and spare mammalian cells given that the outer leaflet of their plasma membrane is dominated by zwitterionic phospholipids (McCafferty et al., 1999; Anantharaman et al., 2010; Mansour et al., 2015; Bolosov et al., 2017; Li et al., 2017; Wu et al., 2017; Kampshoff et al., 2019; Ruden et al., 2019; Sheard et al., 2019; Li et al., 2020; Duong et al., 2021; Xia et al., 2021; Greve and Cowan, 2022; Howell et al., 2022; Zhu et al., 2022). The mechanisms of membrane disruption by HDPs are highly debated, but some consensus exists on a few points (Peschel and Sahl, 2006; Rea et al., 2010; Kong et al., 2012; Mansour et al., 2015; Pérez-Cobas et al., 2015; Kumar et al., 2018; Izoré et al., 2019; Kintses et al., 2019; Mookherjee et al., 2020; Silveira et al., 2021; Torres et al., 2021). First, binding by many membrane-active peptides induces positive membrane curvature strain and thinning by intercalating with the lipid headgroups and expanding the local surface area of the bilayer at constant hydrocarbon density. Second, once the peptides reach a critical threshold concentration, they reorient to insert more deeply and permeabilize the membranes, forcing the cellular/vesicular content to leak out. Structures that have been proposed to induce permeabilization include toroidal pores, carpets, and surface defects. In addition to direct membrane permeabilizing effects, HDPs can induce changes in the physicochemical properties of pathogenic cell membranes, which would indirectly affect the function of their membrane proteins (Wenzel et al., 2014; Omardien et al., 2018). In this regard, P1 was recently shown to lower the threshold at which mechanosensitive channels are activated in *E. coli* model membranes (Comert et al., 2019).

In the fight against enveloped viruses, HDPs present multiple advantages. Their membrane activity spans a broad range of membrane compositions and they are fit to work on protective barriers such as the respiratory tract and skin, as shown from studies with cathelicidins, defensins, histatins, neuropeptide Y, magainins, piscidins, aureins, pleurocidin, and pardaxin (El Karim et al., 2008; Hans and Madaan Hans, 2014; Conlon, 2015; Mahlapuu et al., 2020; Mookherjee et al., 2020). Furthermore, HDPs have dual antimicrobial and immunomodulatory effects, and thus have emerged as a possible answer to the unmet need of a dual-function therapeutic approach to address both infection and inflammation (Beisswenger and Bals, 2005; Gordon et al., 2005; Yount and Yeaman, 2012; Haney and Hancock, 2013; Hilchie et al., 2013; Mansour et al., 2014; Mansour et al., 2015; Boto et al., 2018). Several studies have reported on HDPs that have efficacy *in vitro* and *in vivo* in the context of multiple respiratory viral diseases (Zambrowicz et al., 2013; Uhlig et al., 2014; Mansour et al., 2015; Boto et al., 2018). However, only a fraction of HDPs (6%) have documented antiviral action (Wang et al., 2010; Wang, 2013). A possible reason for this low statistic includes that due to their origin, viral envelopes feature a much lower content of anionic lipids compared to bacterial cell membranes, and therefore favorable electrostatic interactions with cationic HDPs are reduced. It is also the case that a number of viral envelopes are enriched in Chol (Brügger et al., 2006; Chan et al., 2010; Heaton and Randall, 2011; Ivanova et al., 2015).

Chol plays a crucial role in determining the phase behavior of membranes, including the formation of lipid rafts in the plasma membranes of mammalian cells (Brown and London, 1998; Pike, 2003; Tulenko et al., 2007; Simons and Sampaio, 2011; Krause and Regen, 2014; Nicolson, 2014; Lorent et al., 2017). Due to its rigid ring structure, it preferentially interacts with the saturated acyl chains of lipids and prevents their *trans-gauche* isomerization (Krause and Regen, 2014; Yang et al., 2016; Hao et al., 2018). This reduces the dynamics and fluidity of the membrane and increases its order and stability (Mihailescu et al., 2011). In model membrane systems, the equivalent of the lipid rafts is believed to be the liquid ordered (L_o_) phase that, in comparison to the liquid disordered (L_d_) phase, is enriched in Chol and phospholipids with saturated fatty acid chains (Vereb et al., 2003; Engelman, 2005; van Meer et al., 2008; Krause and Regen, 2014; Nicolson, 2014). The higher order and stability that Chol imparts to the L_o_ signifies a protective role against membrane-active peptides, including HDPs (Katsu et al., 1990; Matsuzaki et al., 1995; Epand et al., 2006; Wessman et al., 2008; McHenry et al., 2012; Qian and Heller, 2015). However, some membrane-active peptides are more disruptive when phase-separated (“heterogenous”) L_o_/L_d_ membranes *versus* single-phase (“homogenous”) bilayers are used. They appear to concentrate at domain boundaries in heterogenous membranes (Schön et al., 2008; Blicher et al., 2009; Apellániz et al., 2011; Hao et al., 2018; Nasr et al., 2020; Kumar et al., 2022; Utterström et al., 2022). This interface features curvature strain due to the hydrophobic mismatch between the thicker L_o_ and thinner L_d_, and thus is conducive to defect formation (Brender et al., 2008; Sciacca et al., 2016; Hao et al., 2018). By associating preferentially with this more susceptible region of the membrane, the peptides can achieve enhanced membrane activity. This mechanism has been investigated in the context of several peptide families, including fusogenic peptides, amyloid peptides, and fusion inhibitors (Schön et al., 2008; Blicher et al., 2009; Apellániz et al., 2011; Hao et al., 2018; Nasr et al., 2020; Kumar et al., 2022; Utterström et al., 2022). Still, little is known about its applicability to membrane-active and antiviral HDPs. In-depth mechanistic studies must be performed on these peptides before they can be developed into more potent and selective antiviral agents.

Piscidins are unique templates to accelerate discovery of novel anti-infective therapeutics (Lauth et al., 2002; Noga and Silphaduang, 2003; Lauth et al., 2005; Dezfuli et al., 2012). They are active against many bacteria and viruses, including coronaviruses, methicillin-resistant *Staphylococcus aureus* (MRSA), and *Streptococcus pneumoniae* (*S. pneumoniae*) (Silphaduang and Noga, 2001; Lauth et al., 2002; Noga and Silphaduang, 2003; Menousek et al., 2012; Wang, 2013; Lee et al., 2014; Huang et al., 2015a; Huang et al., 2015b; Lei et al., 2018; Hu et al., 2019; Oludiran et al., 2019). P1, the most widely studied piscidin isoform, has activity against bacterial endotoxin and anti-inflammatory effects. It inactivates multiple classes of enveloped viruses, including the HIV-1 virus, pseudorabies virus, porcine CoV, and channel catfish virus (Chinchar et al., 2004; Wang et al., 2010; Wang, 2013; Lei et al., 2018; Hu et al., 2019). P1 is directly active against pseudorabies and porcine CoV and it is much more effective on the latter than lactoferricin, another important HDP (Lei et al., 2018; Hu et al., 2019). Another advantage of P1 is that it is biocompatible and has favorable pharmacokinetic/pharmacodynamics metrics *in vivo*, including in the context of CoV infections (Chen et al., 2015; Kumar et al., 2017; Lei et al., 2018; Hu et al., 2019). In particular, P1 was safely used in mice infected with the pseudorabies, leading to lower mortality (Hu et al., 2019).

Mechanistic studies focused on the direct action of P1 on pathogenic cells have revealed that it adopts an amphipathic α-helical structure in the membrane-bound state. Upon inserting into the membrane in an orientation parallel to the membrane surface, it disrupts the packing of the phospholipids. As part of this disordering effect, the lipid acyl chains form “baskets” to wrap around the peptide and fill the gap under it. An important consequence of this conformational rearrangement of the acyl chains is a pronounced thinning of the membrane (Perrin et al., 2014; Hayden et al., 2015; Perrin et al., 2015; Mihailescu et al., 2019; Comert et al., 2021; Liu et al., 2023). Prior work has also revealed that P1 induces and leverages membrane heterogeneity to disrupt homogeneous bilayers containing Chol (Comert et al., 2019). Its mechanism of action involves pushing away Chol, leading to lipid demixing (phase separation) and enhanced peptide accumulation in the more disordered region of the membrane.

Notably, the antimicrobial and membrane activities of P1 are associated with its N-terminal region that has a cluster of aromatic residues (e.g., histidines; phenylalanines) and an amino-terminal-copper-and-nickel (ATCUN) motif (Lee et al., 2007; Libardo et al., 2017; Kim et al., 2018; Paredes et al., 2020; Fu et al., 2021). Aromatic residues have been identified to be important for membrane interaction and membrane-remodeling, and thus could point at important design principles to develop new therapeutics that act at membranes (Epand et al., 2005; Apellániz et al., 2011; Zhou and Xu, 2012; Fantini and Barrantes, 2013; Paterson et al., 2017). For instance, Chol can establish favorable π-stacking interactions with aromatic sidechains. Once bound to Chol-containing membranes, P1 can induce positive curvature strain (Perrin et al., 2015). In the context of viral infections, this would counteract the negative spontaneous curvature that characterizes viral fusion intermediates (Risselada, 2017; Shekunov et al., 2023). The capability of P1 to interact and disrupt Chol-containing membranes sets an interesting stage when it comes to investigating how it directly disrupts enveloped viruses.

Here, we demonstrate that the antiviral activity of P1 against SARS-CoV-2 includes direct action on the virus. To better understand the ability of P1 to act on viruses that differ significantly in membrane compositions, we extend the studies previously done with homogeneous L_d_ bilayers. Zwitterionic Chol-containing membrane mimics featuring the L_o_ and L_o_/L_d_ phases are designed. Using complementary biophysical methods, including circular dichroism (CD), solid-state nuclear magnetic resonance (SS-NMR), cryogenic electron microscopy (Cryo-EM), differential scanning calorimetry (DSC), dynamic light scattering (DLS), and machine learning (ML) tools, we investigate how the peptide interacts with and disrupts viral envelope mimics that feature a high Chol content. Through these studies, we reveal novel insights into the mechanism that enables P1, an HDP with antiviral action, to disrupt membranes featuring a broad range of lipid compositions relevant to viral envelopes. This is an important step towards the rational design of broad-spectrum antiviral therapeutics.

## Methods

### Materials

Carboxyamidated P1 (FFHHIFRGIVHVGKTIHRLVTG, MW 2,571) was obtained from the University of Texas Southwestern Medical Center. Fmoc solid-phase peptide synthesis was performed to produce the peptide in the unlabeled and ^15^N-labeled states, as described previously (Chekmenev et al., 2006a; Chekmenev et al., 2006b). The labeled amino acids (^15^N, 98%) were purchased from Cambridge Isotope Laboratories (Tewksbury, MA, United States). Purification of the crude peptide was performed by HPLC as previously reported to yield 98% pure peptide, as characterized by HPLC and mass spectrometry (Chekmenev et al., 2006b; Oludiran et al., 2019). Next, P1 was dissolved in dilute HCl to substitute chloride ions for trifluoroacetate ions. Excess chloride ions were removed by dialysis. The lyophilized peptide was dissolved in nanopure water prior to using it in the studies presented here. The concentrations of the stocks were determined by amino acid analysis performed at the Protein Chemistry Center at Texas A&M (College Station, TX). Metallation of P1 in a stoichiometric ratio with Cu^2+^ was achieved using copper chloride, as described previously (Libardo et al., 2017; Rai et al., 2019; Paredes et al., 2020; Comert et al., 2021; Fu et al., 2021). Reagents such as sodium hydroxide, buffers, salts, EDTA, chloroform, and copper chloride were purchased from Fisher Scientific (Hampton, NH). Calcein dye was obtained from Sigma-Aldrich (Saint Louis, MO). The Millipore MilliQ system (Sigma Aldrich, Saint Louis, MO) was used to obtain Nanopure water.

Lipids were acquired from Avanti Polar Lipids (Alabaster, AL). Non-deuterated lipids were dissolved in deuterated chloroform and concentrations quantified by solution NMR.

### Cell culture

Vero E6 cells (ATCC, CRL-1586, Manassas, VA, United States) were cultured with Eagle’s minimum essential medium (EMEM, Quality Biological, 112–016,101CS, Gaithersburg, MD, United States) supplemented with 10% heat-inactivated fetal bovine serum (FBS, Gibco, Thermo Fisher Scientific, Waltham, MA), 10 U/mL penicillin and 10 μg/mL streptomycin antibiotics (Corning 30–002-CI, Corning, NY, United States), and 2 mM L-glutamine (Corning, 25–005-CI, Corning NY, United States). Vero cells (ATCC, CCL-81, Manassas, VA, United States) were cultured with Dulbecco’s Modified Eagle’s Medium (DMEM, Quality Biological, 112–013,101CS, Gaithersburg, MD, United States) supplemented with 5% heat-inactivated FBS, 10 U/mL penicillin and 10 μg/mL streptomycin antibiotics (Corning 30–002-CI, Corning, NY, United States), and 2 mM L-glutamine (Corning, 25–005-CI, Corning NY, United States). All cell lines were cultivated at 37°C and 5% CO_2_.

### Cell viability assay

Vero E6 cells were seeded at 1.5 × 10^4^ cells per well in 96-well plates and incubated at 37°C and 5% CO_2_ for 24 h. Cells were then treated with 20 μg/mL or 10 μg/mL of P1 for 24 h and cell viability was determined using CellTiter-glo Luminescent Cell Viability Assay (Promega, G7570, Madison, WI, United States) following the manufacturer’s instructions and compared to vehicle control.

### SARS-CoV-2 infections

SARS-CoV-2 (Washington strain 2019-nCoV/USA-WA1/2020) was obtained from BEI Resources (NR-52281) and was used for all infections. Vero E6 cells were seeded at 1.5 × 10^4^ cells per well in 96-well plates 24 h prior to infection. P1 was dissolved in water and diluted in culture media to the indicated concentrations. For direct viral treatment, the virus was diluted to a multiplicity of infection (MOI) of 0.1 in media containing P1 at 10 μg/mL or vehicle control. For pre-treatment and post-treatment infections, cells were pretreated with P1 at 10 μg/mL or H_2_O vehicle control for 1 h at 37°C and 5% CO_2_. Pre-treatment was removed and cells were infected at an MOI of 0.1 in culture media for 1 h at 37°C and 5% CO_2_. After infection, inoculum was removed and post-treatment, P1 at 10 μg/mL or H_2_O vehicle, was applied. Treated inoculum was incubated at 37°C and 5% CO_2_ for 1 h and then applied to cells for 1 h. After infection, inoculum was removed, and culture media was applied to the cells. For infections combing pre-treatment, direct viral treatment, and post-treatment with P1, the above steps with P1 were combined. For post-treatment only infections, cells were infected at an MOI of 0.1 for 1 h. After infection the inoculum was removed and media containing P1 at 10 μg/mL or H_2_O vehicle control was added to the cells and incubated at 37°C and 5% CO_2_. After 24 h post infection (hpi), viral supernatants were collected and used immediately for assays or stored at −80°C.

### Plaque assay

Vero cells were plated in 12-well plates at a density of 2 × 10^5^ cells per well and incubated for 24 h. Infection supernatants were serially diluted to 10^−6^ in culture media and overlaid on cells for 1 h. Cells were covered with Eagle’s Minimum Essential Medium (without phenol red (Quality Biological 115–073-101, Gaithersburg, MD, United States), supplemented with 5% FBS, 1% nonessential amino acids, 1 mM sodium pyruvate (VWR, 45,000–710, Dixon, CA, United States), 2 mM L-glutamine, 20 U/mL penicillin, and 20 μg/mL streptomycin) with 0.6% agarose (ThermoFisher, 16,500,100, Waltham, MA, United States). At 48 hpi, cells were fixed with 10% formaldehyde (ThermoFisher, F79P-4, Waltham, MA, United States) for 1 h. Medium was removed, wells were washed with diH2O and stained with a 1% crystal violet (ThermoFisher, C581-25, Waltham, MA, United States) and 20% ethanol solution (ThermoFisher, BP2818-4, Waltham, MA, United States).

### Circular dichroism

The secondary structure of P1 interacting with the L_o_ and L_o_/L_d_ viral envelope mimics was accomplished by CD in potassium phosphate buffer (5 mM, pH 7.4). LUVs were made from lipid films, as described previously (Chekmenev et al., 2006b; Paredes et al., 2020). Briefly, the films were made using DPPC, POPC, DPPE, POPE, and Chol (Avanti Polar Lipids, Alabaster, AL) mixed in chloroform in the amounts needed to form the L_o_ or L_o_/L_d_. The solvent was evaporated under N_2_ gas and the films dried under vacuum overnight. The films were then hydrated with phosphate buffer (5 mM, pH 7.4), subjected to freeze-thaw cycles, and extruded using an extruder (Avanti Polar Lipids, Alabaster, AL) and 0.1 µm size membranes (Whatman, Florham Park, NJ). The vesicles were then diluted to 3 mM. Each spectrum was performed at fixed peptide concentration (20 µM). P1 was added to reach various P/L ratios, ranging from 1:20 to 1:200. CD spectra were acquired at 298 K on a Jasco J-1500 spectrometer (Jasco Analytical Instruments, Easton, MD). Experimental parameters included a wavelength range of 190–260 nm, scan speed of 100 nm/min, 1 nm bandwidth, and number of scans equal to four. The samples were run in duplicates. A blank containing buffer and lipids but no peptide was recorded and subtracted from the signal obtained for the peptide-containing sample, as needed to account for the background signal. Peptide helicity was obtained using the molar ellipticity measured at 222 nm and assuming an ellipticity of −32,000 deg·cm^2^/dmol for an ideal α-helix (Lee et al., 2007).

### Permeabilization via dye leakage release from large unilamellar vesicles

The permeabilization assays were performed as previously described (Mihailescu et al., 2019; Liu et al., 2023). Briefly, for each lipid composition, either L_o_ or L_o_/L_d_, a film was made in round bottom flask using DPPC, POPC, DPPE, POPE, and Chol (4 µmol total of lipids) mixed at a desired molar ratio in chloroform. After evaporating the organic phase under N_2_ gas, the film was lyophilized overnight. Next, the film was hydrated with 300 µL of 80 mM calcein dye (pH 7.4) before undergoing three freeze-thaw cycles and extrusion through a mini extruder (Avanti Polar Lipids) using a 0.1 µm polycarbonate membrane. Free dye was removed using a size-exclusion column with a Sephadex G-50 resin stationary phase run with HEPES buffer (50 mM, 100 mM NaCl, 0.3 mM EDTA, pH 7.4). The exact lipid concentration of the LUVs was obtained using a Total Phosphorus Assay (Chen et al., 1956). They were then diluted to a final concentration of ∼35 μM, and distributed in a 96-well plate using 180 µL per well. Next, 20 µL of P1 solution was added to each well in concentrations needed to result in specific peptide-to-lipid ratios (P/L) in the wells to bracket P/L = 1:2 and 1:2048 based on the serial dilution of the P1 stock. Each P/L value was run in triplicate and at least three independent assays were run. Positive and negative controls featured 20 µL of 1% Triton-X detergent or 20 µL nanopure water instead of the peptide solution. Each plate was shaken for 1 hour at 45 °C (or 30 °C) and allowed to cool for 20 min before recording the fluorescence intensity using a BioTek SynergyH1 plate reader (BioTek, Winooski, VT). An excitation wavelength of 490 nm and an emission wavelength of 520 nm was used for calcein. Fractional peptide-induced leakage was obtained using the equation:

$$\%\;leakage=\frac{I_{x}-I_{0\%}}{I_{100\%}-I_{0\%}}$$

where I_x_ is the fluorescence intensity in a well with a particular peptide concentration, I_100%_ is the average intensity in the positive-control wells (100% leakage) and I_0%_, is the average intensity in cells with the negative control (0% leakage). The fractional leakage was then plotted against lipid-to-peptide ratio (L/P) and fitted in GraphPad Prism using the Hill equation to identify the EC_50_ or P/L at which half of the vesicles were lysed (Sani et al., 2014). The 95% confidence interval (CI) was obtained from the program.

### Preparation of large unilamellar vesicles for Cryo-EM

Lipid films of samples featuring the L_o_ or L_o_/L_d_ were made using the needed molar ratio of DPPC, POPC, DPPE, POPE, and Chol in chloroform. After evaporating the solvent and vacuuming overnight, the films were hydrated with potassium phosphate buffer (5 mM, pH 7.4), diluted, and extruded through a mini extruder (Avanti Polar Lipids) using 0.1 µm polycarbonate membrane (Whatman, Florhan Park, NJ). Piscidin was added in varying amounts to reach specific P/L ratios ranging from 1:20 to 1:200. To confirm the size of the LUVs made for Cryo-EM experiments, we used Dynamic Light Scattering (DLS). This involved transferring 540 µL of vesicles into 2 mL Eppendorf tubes. The particle size distribution of the samples was measured at room temperature using a Nicomp N3000 Submicron Particle Sizer (Particle Sizing Systems). Some peptide-containing samples were also tested. In this case, we used 60 µL of peptide solution to reach a specific P/L. For lipid-only samples, 60 µL of nanopure water were used instead of peptide solution.

### Cryo-EM experiments

Cryo-EM experiments were performed at the New York Structural Biology Center (NYSBC). Before cryo grids preparation, the samples were warmed up to 50 °C for 5–10 min. Then 4 μL of the solution was immediately added to freshly plasma cleaned (Gatan Solarus plasma cleaner, 75% argon/25% oxygen atmosphere at 15 Watts for 7 s) Au R1.2/1.3 300-mesh (UltrAuFoil^®^) grids. Grids were blotted for 4 s or 5 s after a 3 s pre-blotting time, then plunge-frozen in liquid ethane using Vitrobot Mark IV with the chamber maintained at 20°C and 100% humidity. Cryopreserved grids were stored in liquid N_2_ until use. Image collection was accomplished at ∼2 μm under focus on a Thermo Scientific™ Glacios™ Cryo-Transmission Electron Microscope (Cryo-TEM) operated at 200 kV equipped with a Falcon3 operated in linear mode. Data collection was performed in the automatic data collection software Leginon (Suloway et al., 2005) at 2.5 Å or 1.2 Å per pixel. The dose was fractionated over 30 raw frames and collected with the total electron dose around 31.0 e^–^/Å^2^. Individual frames were aligned and dose-weighted with MotionCor2 (Zheng et al., 2017).

### Cryo-EM data analysis

#### Membrane segmentation and contour extraction

Membranes were segmented in micrographs collected for each liposome preparation using an implementation of the MemNet algorithm available on GitHub. MemNet uses convolutional neural networks to annotate membranes in micrographs using a U-Net-like neural network architecture. The network was trained and evaluated on a subset of micrographs collected in this study in which membranes were manually annotated using the VGG Image Annotator (Dutta and Zisserman, 2019) and a second set of micrographs containing liposomes provide by Fred Heberle (University of Tennessee, Knoxville).

The neural network outputs pixel masks containing the probability, for each pixel, that it is within a lipid bilayer. Contours were extracted from these by identifying points tiling the positive segmented membrane regions with 40 Å spacing, using the “predict_contours.py” script. Membrane fragments containing fewer than 10 coordinates were removed. Contours were then split into linear fragments and smoothed using a spline fit, the “smooth_contours.py” script. Linear fragments were identified by finding contiguous chains of points. Because these micrographs often contain tightly packed, intersecting, or overlapping membranes for which we could not resolve the full contour of single liposomes, we broke liposomes boundaries into contiguous linear fragments by removing points which could have more than two neighbors (intersecting points) for downstream analysis.

#### Membrane curvature analysis

In order to estimate the curvature of the membranes at each point in the membrane contours, we first estimated the curve tangent and normal vectors using finite differences. Let the *x* and *y* coordinates of the curve be parameterized in terms of an auxiliary variable, *t*, which denotes the distance along the curve from an arbitrary starting point. The tangent vectors to the curve are $\frac{dx\left(t\right)}{dt},\frac{dy\left(t\right)}{dt}$ which we refer to as $x^{\prime }$ and $y^{\prime }$ for simplicity. The normal vectors to the curve are $\frac{d^{2}x\left(t\right)}{dt^{2}},\frac{d^{2}y\left(t\right)}{dt^{2}}$ which we refer to as $x^{\prime \prime }$ and $y^{\prime \prime }$ for simplicity. The curvature at some point is then defined as

$$\kappa =\frac{x^{\prime \prime }y^{\prime }-\;y^{\prime \prime }x^{\prime }}{{\left(x^{\prime }x^{\prime }+y^{\prime }y^{\prime }\right)}^{\frac{3}{2}}}$$

where κ is the curvature in 1/Å. This is calculated using the “analyze_contours.py” script.

#### Membrane width analysis

Membrane thicknesses were estimated by first linearizing each membrane fragment along its contour to find a centered, linearized membrane profile, similar to Heberle et al. (Heberle et al., 2020) For each point along the membrane contour, these membrane profiles were estimated for 20 nm high and 5 nm wide slices through the membrane. These membrane profiles were then lightly smoothed using a Gaussian filter along the length dimension of the linear profile with a small standard deviation of 1.5 pixels.

To estimate the membrane widths, we first found the bilayer peak signals by fitting an 8^th^ order polynomial to the signal intensities for each linearized membrane slice, using the polyfit algorithm in numpy (Harris et al., 2020). We then found the locations of the peaks by finding the smallest critical points in the fit polynomials. The width of the membrane at each location was then defined as the distance between these peaks in Å.

#### Statistical analysis of membrane biophysical properties

We compared the biophysical properties of the membranes across liposome preparations spanning a range of P/L ratios and L_o_ and L_o_/L_d_ phases. For each condition, we extracted membrane contours and estimated membrane curvatures and widths as described above. In total, we analyzed 2,707,744 5 nm membrane fragments across 10 conditions. This added up to more than 1.3 cm of contiguous membrane analyzed.

**Table:** 

Phase	Peptide:Lipid	Counts
L_o_	0:1 (lipid blank)	378,864
L_o_	1:500	62,173
L_o_	1:120	150,562
L_o_	1:40	483,155
L_o_/L_d_	0:1 (lipid blank)	20,791
L_o_/L_d_	1:2000	42,335
L_o_/L_d_	1:500	128,198
L_o_/L_d_	1:120	1,074,657
L_o_/L_d_	1:40	161,903
L_o_/L_d_	1:20	205,106
	Total	2,707,744

We examined the differences in the mean curvatures and widths between conditions and assessed their statistical significance using a two-sample Student’s t-test. To understand the effects of ligand concentration and phase on membrane curvature, we also fit ordinary least squares models to predict the observed curvatures from the log of the P/L ratios, phases, and ratio-phase cross terms using the “statsmodels” python package (Seabold and Perktold, 2010).

### Preparation of oriented samples for solid-state NMR

Oriented samples were made to characterize the structural and orientational features of P1 that had been exposed to the L_o_/L_d_ viral envelope mimics, and to determine how P1 affects the phase behavior of these model membranes. Aligned phospholipid preparations were achieved using a protocol previously established (Chekmenev et al., 2006a; Perrin et al., 2014). Briefly, the samples were made using the L_o_/L_d_ mixture as viral membrane mimics, i.e. 20:10:12:33:25 DPPC/DPPE/POPC/POPE/Chol. For ^2^H detection, 5 mg of DPPC-d62 was incorporated. For the sample containing P1, after the lipids were mixed in chloroform, P1 dissolved in trifluoroethanol was added at P/L = 1:100. About 2 mg of ^15^N-V_10_G_13_I_16_ P1 was used. After co-dissolving all of the components, the solvent was evaporated using nitrogen gas and lyophilized overnight. The next day, the lipids were hydrated with 1 mL of Bis-Tris buffer (3 mM, pH 7.4). The mixture was incubated overnight at 40 °C and then spread on thin glass slides (dimensions 5.7 × 12 × 0.03 mm^3^ from Matsunami Trading Co., Japan). Each sample was then allowed to equilibrate in humidity chamber (>90% using a saturated potassium sulfate solution). The slides were stacked after hydrating at 42% by weight using ^2^H-depleted water. The stacked samples were placed in a glass cell (Vitrocom Inc., NJ), sealed with beeswax, and incubated at 45 °C until homogeneously hydrated.

### Preparation of unoriented samples for solid-state NMR

Unoriented samples were prepared using a procedure similar to that used for oriented samples. Deuterated DPPC-d62 was weighed out and mixed in a round-bottom flask with appropriate amounts of non-deuterated lipids to form the L_o_ phase, i.e. 40:18:3:6:33 DPPC/DPPE/POPC/POPE/Chol. The solvent was evaporated, and the film dried on a lyophilizer overnight. The film was then hydrated with Bis-Tris buffer and underwent freeze-thaw cycles to ensure distribution of buffer. The resulting suspension was incubated overnight at 40°C. The suspension was then transferred to a small glass vial and lyophilized. The dry lipid was transferred to a sample tube and hydrated to 42% (v/w) with deuterium-depleted water, so that the final BisTris buffer concentration matched that of the oriented sample. To ensure complete removal of HOD that would give rise to ^2^H NMR signal, the sample was lyophilized at least 4 h, rehydrated to 42% with the ^2^H depleted water, and sealed. The sample was then incubated for 24 h before NMR analysis.

### ^2^H solid-state NMR

^2^H spectra were collected at the National High Magnetic Field Laboratory (NHMFL) on a 600 MHz wide-bore magnet equipped with a Bruker Avance I console where the ^2^H Larmor frequency is 92.12 MHz. Parameters for the quadrupolar echo pulse sequence included a 3.0 µs 90° pulse on ^2^H, and an echo time of 20 µs. The FIDs were recorded with a dwell time of 2 µs for an acquisition time of 4.096 ms. Samples were first run at 50°C to allow for annealing, then run from 10°C up to 50°C in steps of 5°C. A spectrum was also obtained at 42°C between the 40°C and 45°C spectra. Each sample was allowed to equilibrate at each temperature before 4,096 and 1,536 scans for the oriented and unoriented samples, respectively, were averaged with a recycle delay of 1 s. The data were processed in Topspin (Bruker, Billerica, MA) with 100 Hz of exponential linebroadening and referenced to D_2_O at 0 kHz. The data from the unoriented L_o_ sample were de-Paked in NMRPipe (Delaglio et al., 1995) using the dePake macro detailed in Sani et al. (Sani et al., 2013). The FID was left-shifted to the echo maximum before applying a sinebell function, zero-filling, and dePakeing by weighted fast Fourier transform. Basic peak detection within NMRPipe was used to identify the position of resonances and yield the quadrupolar splittings.

### ^1^H solid-state NMR

^1^H spectra were acquired at the NHMFL on a 600 MHz wide-bore magnet equipped with a Bruker Avance I console where the ^1^H Larmor frequency is 600.13 MHz. A pulse sequence that suppresses the background signal from the probe was implemented (Wang et al., 2021). Samples packed in 3.2 mm rotors with caps containing O-rings (Revolution NMR, Fort Collings, CO) were spun at 10 kHz. Parameters included a 4.0 µs 90° pulse, and a recycle delay of 2 s. Samples were first run at 50°C to allow for annealing, then run from 10°C up to 55°C in steps of 5°C, with a spectrum at 42°C collected between the 40°C and 45°C spectra. The data, corresponding to 32 scans, were processed with no window function and referenced to the methylene signal at 1.30 ppm (Veatch et al., 2004; Polozov et al., 2008) TopSpin (Bruker, Billerica, MA) was used to process the data. Peak picking was used to determine the intensity of the methylene peak at 1.30 ppm. The peak width at half height was determined by measuring from the picked peak to the half-height on the left as the right side was convoluted in spectra.

### ^15^N solid-state NMR

2D SAMPI4 (Nevzorov and Opella, 2007) spectra were recorded on a 600 MHz wide bore Bruker instrument with Avance I console at the NHMFL where the ^1^H and ^15^N frequencies were 600.13 and 60.82 MHz, respectively. The temperature was set to the indicated temperature ±0.1°C. For each spectrum, a recycle delay of 4 s and 32 t_1_ increments with 2048 transients each were used. The pulse sequence used a contact time of 810 µs, cross-polarization field of 50.0 kHz, and ^1^H decoupling of 62.5 kHz (using PWTPPM) (Fu, 2009). Data were processed using Topspin with Gaussian window function (LB = - 50 Hz, GB = 0.1) in the ^15^N dimension and (LB = - 100 Hz, GB = 0.1) in the ^1^H-^15^N dimension. The ^15^N chemical shifts were referenced to a saturated solution of ammonium sulfate set at 26.8 ppm with respect to ammonia.

### Differential scanning calorimetry

#### Sample preparation

Lipids in chloroform (Avanti Polar Lipids, Alabaster, Alabama) were used for the sample preparations. Individual POPE and DPPC vesicles in water were obtained by separating aliquots of lipid in chloroform in glass vials, removing the organic solvent under a stream of nitrogen gas and placing the samples under vacuum for 2 h. The dry films were reconstituted in water with repeated agitation, followed by 10 min bath sonication at 50°C. The vesicles in water were stored for several hours (less than 24 h) at 4°C before the DSC measurements. Homogenous mixtures of POPE/DPPC at (1:1 M ratio) or POPE/DPPC/Chol (1:1:0.5) were prepared as above by first co-dissolving the lipid and Chol in chloroform in the desired proportions. Sample with P1 peptide were obtained by adding the peptide to lipid vesicles in water just before the measurement.

#### DSC measurements

Differential scanning calorimetry (DSC) measurements were made on a VP-DSC microcalorimeter (MicroCal Inc., Northampton, MA). The samples were degassed and equilibrated at the starting scan temperature for 5 min, just before measurements. At least three cycles of heating-cooling curves were collected, at a scan rate of 30 °C/hour and in the range of 12°C to 95°C. There was a 5 min equilibrating period prior to starting the experiment and a delay of 5 min between sequential scans to allow for thermal equilibration. Samples with and without peptides in the same category were always prepared from the same stock of vesicle in water, and measured one after the other, following the same measurement routine. DSC curves were analyzed by Origin, version 7.0 (OriginLab Corporation). The scans were corrected by subtracting the background, normalizing to lipid concentration, and applying baseline correction.

## Results

### Piscidin 1 inhibits SARS-CoV-2 in cell cultures

**FIGURE 1:**
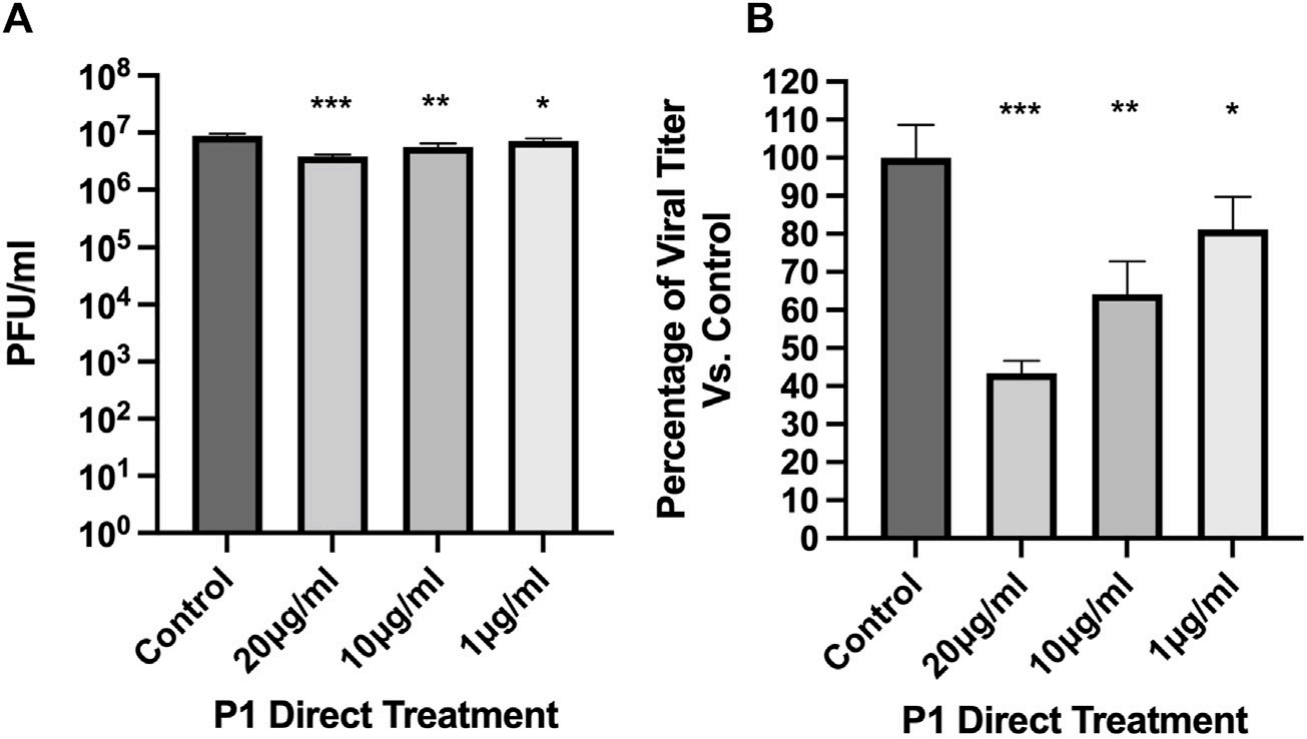
Direct antiviral effects of Piscidin 1 on SARS-CoV-2. The plaque assay was employed on cultured Vero E6 cells to quantify the amount of SARS-CoV-2 infectious titer after direct treatment with Piscidin 1 (P1). The virus was diluted to an MOI of 0.1 in media containing P1 at the indicated concentrations or vehicle control. Cells seeded at a density of 1.5 × 10^4^ per well were then exposed to the virus. **(A)** Data are reported as the average plaque forming units (Avg PFU/mL) over triplicates; *p > 0.05; **p > 0.01; and ***p > 0.001. **(B)** The viral titer is plotted as a percentage of the H_2_O control. P1 demonstrated inhibitory effect on SARS-CoV-2. Under the conditions tested, P1 was more effective than Brilacidin used as a reference antiviral peptide (Supplementary Figure S1).

The potential for piscidins to exert an antiviral activity against SARS-CoV-2 was assessed using Vero E6 cells as an infection model. The Cell Titer Glo assay was used to determine cell viability 24 h post infection (hpi). For the initial assessment, we aimed at determining if the peptide could directly inhibit the ability of the virus to establish a productive infection in cells. For this purpose, the virus (Washington strain 2019-nCoV/USA-WA1/2020) was directly exposed to piscidin or vehicle control before the cells were infected. After 24 hpi, plaque assays were performed to determine if the peptide inhibited viral infection. As shown in Figure 1, P1 at 20.0, 10.0, and 1.0 μg/mL inhibited viral titers by 56.6%, 35.8%, and 18.9% respectively. This indicates that the piscidin peptide exerts a direct inhibitory effect on the ability of the virus to establish a productive infection.

**FIGURE 2:**
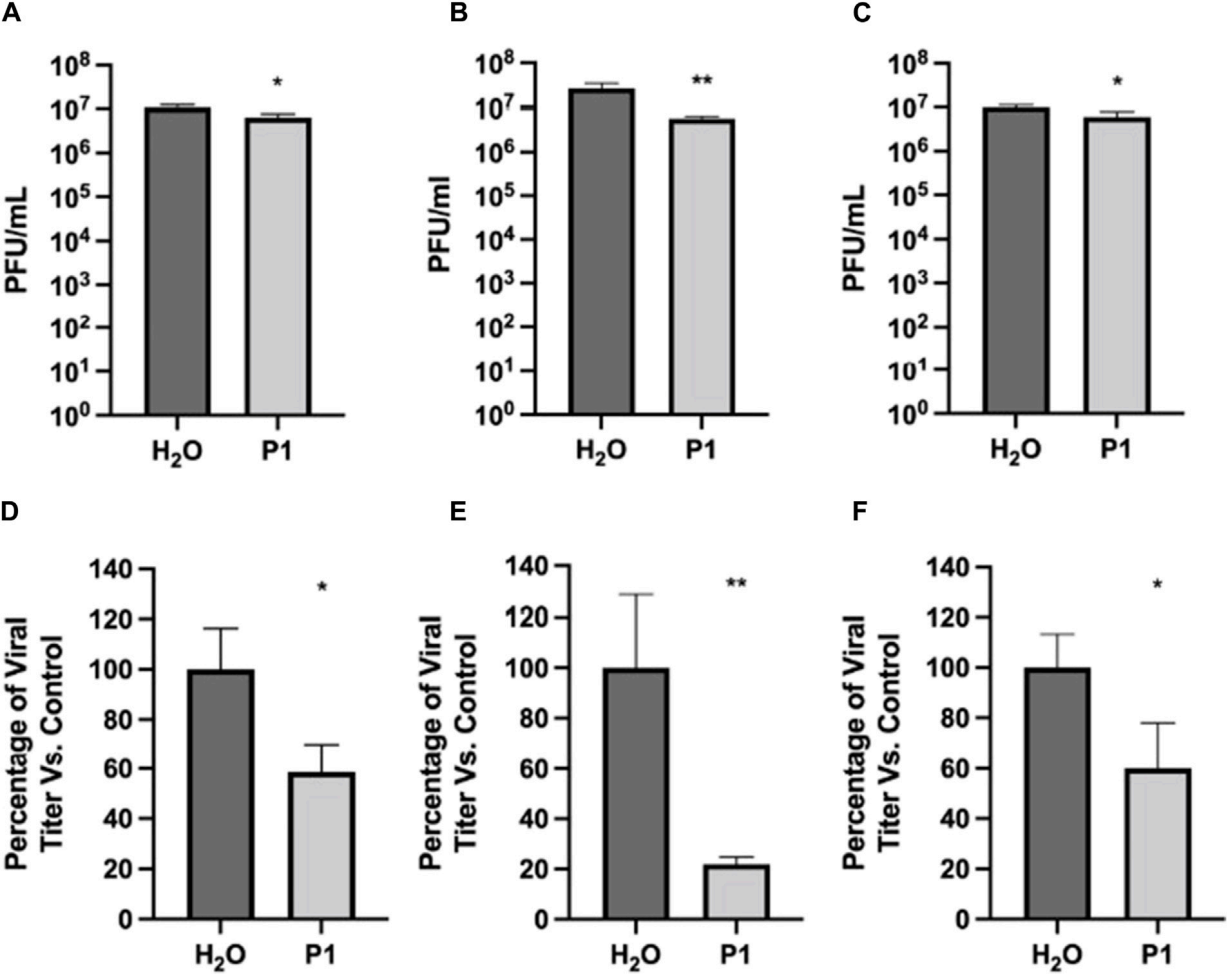
Antiviral effects of Piscidin 1 on SARS-CoV-2 as a function of treatment type. The plaque assay was used on Vero E6 cells to quantify the amount of SARS-CoV-2 infectious titer. Cells were seeded at a density of 1.5 × 10^4^. Infection was done with SARS-CoV-2 at an MOI of 0.1. Three treatment types were tested to complement the direct treatment data in Figure 1. **(A–C)** Viral titer are reported as the average plaque forming units (Avg PFU/mL) over triplicates; *p > 0.05 and **p > 0.01. **(A)** Pre- and post-treatment assay: the cell were pretreated for one hour with peptide at 10 μg/mL; the peptide was removed during infection by the virus and added again after infection. **(B)** Pre-, direct-, and post-treatment assay: the peptide P1 at 10 μg/mL was present at all steps. **(C)** Post-treatment assay: P1 at 10 μg/mL was added only after infection with SARS-CoV-2 had been completed. **(D–F)**: Same **(A–C),** respectively, but as a percentage of the H_2_O control.

Next, pre-treatment and post-treatment were used to determine if post-entry mechanisms were also inhibited by P1. As shown in Figure 2, pre- and post-treatment with 10.0 μg/mL of P1 reduced SARS-CoV-2 viral titer by 41.5% compared to the vehicle control. Similarly, post-treatment alone reduced viral titers by 40.0%. Combining pre-treatment, direct viral treatment, and post-treatment showed the highest inhibition at 78.1% compared to the vehicle control. This suggests that continual treatment with P1 is characterized by a concentration to reach 50% viral inhibition (EC_50_) that is on the order of 5 μg/mL (2 µM). This is stronger than the direct viral treatment described above since the EC_50_ in that case appeared to be on the order of 20.0 μg/mL (8 µM). Overall, these assays indicate that P1 can inhibit the infection potential of the virus in two ways, by directly disrupting the viral particles and indirectly influencing the post-entry infection mechanisms of the virus in cells.

The effectiveness of P1 was compared to that of other peptides, such as the copper-bound form of P1 (P1-Cu) and the piscidin isoforms P3 and TP4, to compare its effectiveness, as shown in Supplementary Figure S1. Brilacidin is a peptidomimetic which we have already published as exerting an antiviral activity against SARS-CoV-2 in this infection model and was used as a positive comparison control (Bakovic et al., 2021). In this case, the pre-treatment strategy was used on Vero cells and each peptide was added at a concentration of 10.0 μg/mL. No apparent cell toxicity was noted following peptide treatment as compared to the vehicle-alone control (data not shown). The data in Supplementary Figure S1 demonstrate that P1 and P3 peptide treatment resulted in a statistically significant decrease as compared to the vehicle-alone control, with P1 demonstrating greater inhibition (>90%) than P3. The extent of inhibition observed with P3, HR9 (histidine-rich nona-arginine) and TP4 was comparable. The conjugation of copper decreased the observed inhibitory potential of both P1 and P3 in a comparable manner. Cumulatively, the data support the antiviral potential of P1 and P3 piscidins against SARS-CoV-2 in cell culture. They also show that the peptides have a direct effect on the virus. Previously, P1 was demonstrated to be active *in vivo* on pseudorabies and porcine CoV (Lei et al., 2018; Hu et al., 2019). It is also inhibitory to HIV-1 *in vitro* (Wang, 2013). Since these viruses have a lipid envelope and piscidin is membrane active, we performed biophysical experiments on viral envelope mimics to gain further insight into the mechanism of direct inhibition.

### Design of viral envelope mimics

As explained in the introduction, enveloped viruses acquire their lipids from host cells, but viral envelopes have lipid compositions that vary from virus to virus and differ from the host plasma membrane. P1 is active on viruses that feature a broad range of lipid compositions, especially with regard to Chol, a lipid that strengthens membranes. It was previously shown to be active on HIV-1 (Wang et al., 2010; Wang, 2013), which has a high Chol content in its envelope (Brügger et al., 2006). The reported EC_50_ was 2.1 µM (5.4 μg/mL), which is similar to the EC_50_ observed here against SARS-CoV-2, a virus that has a low Chol content (Saud et al., 2022). Based on their assembly and egress pathway, the low Chol level also applies to the porcine CoV and pseudorabies that P1 directly inhibits (Hogue et al., 2014; Schoeman and Fielding, 2019; Ghosh et al., 2020). Through our previous studies, we showed that P1 permeabilizes both anionic and zwitterionic L_d_ bilayers. Membranes tested contained lipids such as PC, PE, and Chol, which are relevant to the envelopes of the viruses that P1 inhibits. However, the effect of P1 has not been characterized on the L_o_ and L_o_/L_d_ bilayers that are relevant to dangerous viruses such as HIV-1 and Influenza A (Brügger et al., 2006; Ivanova et al., 2015). To fill this gap and to extend previous studies beyond the L_d_, we designed Chol containing membranes featuring the L_o_ and L_d_.

In preparing bilayers containing the L_o_ and L_d_, we opted for a membrane composition that best represented multiple viruses, including HIV-1 and Influenza A (Brügger et al., 2006; Ivanova et al., 2015). In addition to containing a large amount of Chol, their membranes have an outer leaflet that is enriched in zwitterionic lipids (Brügger et al., 2006). To represent the main lipid headgroups present in these viruses, we used phosphoethanolamine (PE) and phosphocholine (PC), which are also the dominant species in plasma membranes of host cells (Brügger et al., 2006; Ivanova et al., 2015; Lorent et al., 2020). In terms of acyl chains, we used saturated (palmitoyl) and unsaturated (oleoyl) fatty acids, as needed to be able to form both the L_o_ and L_o_/L_d_ phases in the presence of Chol. Table 1 lists the specific lipids and the molar ratios used to generate L_o_ and L_o_/L_d_ systems.

**TABLE 1:** Composition of model membranes (mol fraction).

Viral envelope mimics	L_o_/L_d_	L_o_
%DPPC	20	40
%DPPE	10	18
%POPC	12	3
%POPE	33	6
%Chol	25	33

DPPC, dipalmitoylphosphatidylcholine; DPPE, dipalmitoylphosphatidylethanolamine; POPC, palmitoyloleoylphosphatidylcholine; POPE, palmitoyloleoylphosphatidylethanolamine; and Chol, cholesterol.

### Characterization of L_o_/L_d_ and L_o_ viral envelope mimics exposed to P1 by solid-state NMR

**FIGURE 3:**
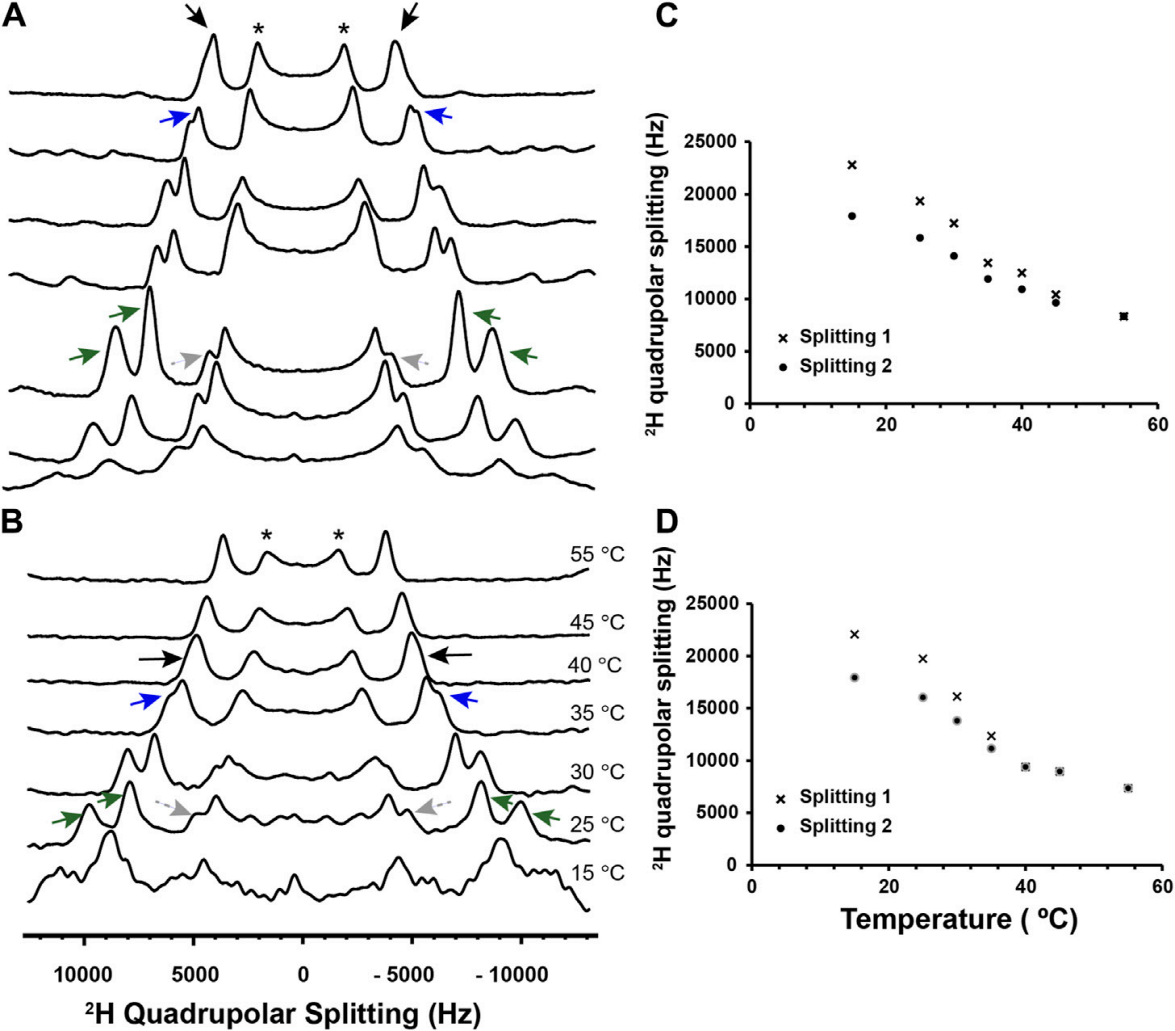
^2^H solid-state NMR of L_o_/L_d_ viral envelope mimics in the absence and presence of P1. Multilamellar L_o_/L_d_ vesicles prepared with 20:10:12:33:25 DPPC/DPPE/POPC/POPE/Chol were studied by ^2^H static solid-state NMR at different temperatures in the absence **(A)** and presence of P1 at P/L = 1:100 **(B)**. For ^2^H detection of the phospholipid methyl region, deuterated DPPC (d62) was incorporated in vesicles that were mechanically aligned on glass plates. Quadrupolar splittings arising from deuterated methyl groups give rise to signals that are centered at 0 kHz. Throughout the temperature range, some residual signal from unoriented lipids is present and indicated with an asterisk. At 55°C, the L_o_ and L_d_ phases are mixed, with DPPC-d62 giving rise to a single quadrupolar splitting (black arrows). As the temperature decreases, the onset of demixing occurs at T_low_, and DPPC-d62 gives rise to broadened peaks that start separating (blue arrows). Dropping the temperature further enables resolved signals to appear for the L_o_ and L_d_ phases. More specifically, the methyl groups of DPPC-d62 in the L_o_ phase give rise to two distinct outer splittings (green arrows). In the L_d_ phase, the lipid produces a single splitting, which is smaller and indicated using gray dashed arrows. It is not fully resolved from the signals arising from the unoriented lipids in the same spectral region. The ^2^H quadrupolar splittings for the L_o_ phase were extracted from the spectra and plotted as a function of temperature for the lipid-only sample **(C)** and sample with P1 **(D)**. Splittings 1 and 2 correspond to the larger and smaller of the L_o_ splittings (green arrows in A and B), respectively. In the presence of P1, the onset of large-scale demixing occurs at T_low_ = 35°C ± 5 °C while it is 45°C ± 5 °C in the lipid-only sample. Thus, the peptide extends the range over which the lipid phases are miscible. The ability of P1 to alter the stability of the L_o_ is confirmed by ^1^H MAS experiments presented in Supplementary Figures S4, S5.

As a first step with the viral envelope mimics, we leveraged ^2^H static SS-NMR to characterize their phase behavior and confirm the presence of the L_o_. To achieve high resolution, oriented lipid bilayers were made and DPPC-d62 was used as the ^2^H reporter. Figure 3A and Supplementary Figure S2 show ^2^H SS-NMR spectra collected at different temperatures to monitor the phase behavior. At the high temperature of 55°C, the methyl region of the ^2^H spectrum displays a single quadrupolar splitting (distance between the two black arrows at the top of Figure 3). This indicates that DPPC-d62 exists in a bilayer that is well mixed, and thus above its miscibility point. When the temperature is decreased to 45°C, multiple splittings start appearing, indicating the onset of demixing and the co-existence of the L_o_ and L_d_. Essentially, the rate at which the DPPC-d62 exchanges between the L_o_ and the L_d_ phases is slow enough on the time scale of the ^2^H NMR experiments that their splittings are becoming resolved from each other (Veatch et al., 2004). The outer splittings, which are well resolved at 30 °C (green arrows in Figure 3) are 17.2 and 14.1 kHz, clearly corresponding to the *sn*-1 and *sn*-2 methyl groups of DPPC-d62 in the L_o_ phase (Veatch et al., 2004). The same chains in the L_d_ phase give rise to the inner splitting (8.4 kHz, gray arrows in Figure 3). As previously explained by the Gawrisch lab, the difference between the inner and outer splittings indicate that large L_o_ domains (>> 160 nm) are present, and thus the samples are experiencing large-scale demixing (Veatch et al., 2004). The onset temperature for this demixing is referred to as T_low_. Using a similar approach, we also investigated the L_o_ system. As displayed in Supplementary Figure S3, two large quadrupolar splittings (e.g.,; 19.5 and 15.4 kHz at 30 °C) are detected, and therefore the L_o_ is present throughout the temperature range. Overall, the signals observed by solid-state NMR confirm that the chosen lipid compositions form the expected L_o_/L_d_ or L_o_ phases.

In the presence of P1 at a peptide-to-lipid ratio (P/L) of 1:100 (Figure 3B), the large-scale demixing of the L_o_/L_d_ occurs at 35°C ± 5°C, which is significantly lower than in the neat bilayer. Thus, P1 exerts a mixing effect on the L_o_ and L_d_, forcing them to mix at a lower temperature than in the neat membrane. In parallel to these ^2^H NMR experiments, the viral envelope mimics were also subjected to magic angle spinning (MAS), the NMR method commonly used to detect the high-resolution ^1^H signals of unoriented samples. As shown in Supplementary Figure S4, the intensity of the central band for the methylene signals from the lipid acyl chains keeps increasing throughout the temperature range, indicating that the miscibility point, T_mix_, has not been reached by 55 °C. However, in the presence of P1 (Supplementary Figure S5), a plateau is achieved at T_mix_ = 40°C ± 5°C, showing that the peptide depresses the stability of the L_o_, in agreement with the ^2^H data. We note that the ^2^H experiments detect large-scale demixing, explaining why its onset occurs at a lower temperature than T_mix_ (Veatch et al., 2004).

### CD-monitored binding of P1 to L_o_/L_d_ and L_o_ viral envelope mimics

Next, the secondary structure of P1 was investigated in the presence of the viral envelope mimics. P1 is unstructured in aqueous solution while it adopts an α-helical structure bound to membranes (Chekmenev et al., 2006b; Perrin et al., 2014; Hayden et al., 2015; Paredes et al., 2020). CD spectroscopy readily resolves these two states, and thus provides a useful tool to determine the secondary structure of P1 when it is exposed to viral envelope mimics containing the L_o_ and L_o_/L_d_. Large unilamellar vesicles were made with a diameter of 0.1 µm to mimic the small size of virions (Lenard and Compans, 1974).

**FIGURE 4:**
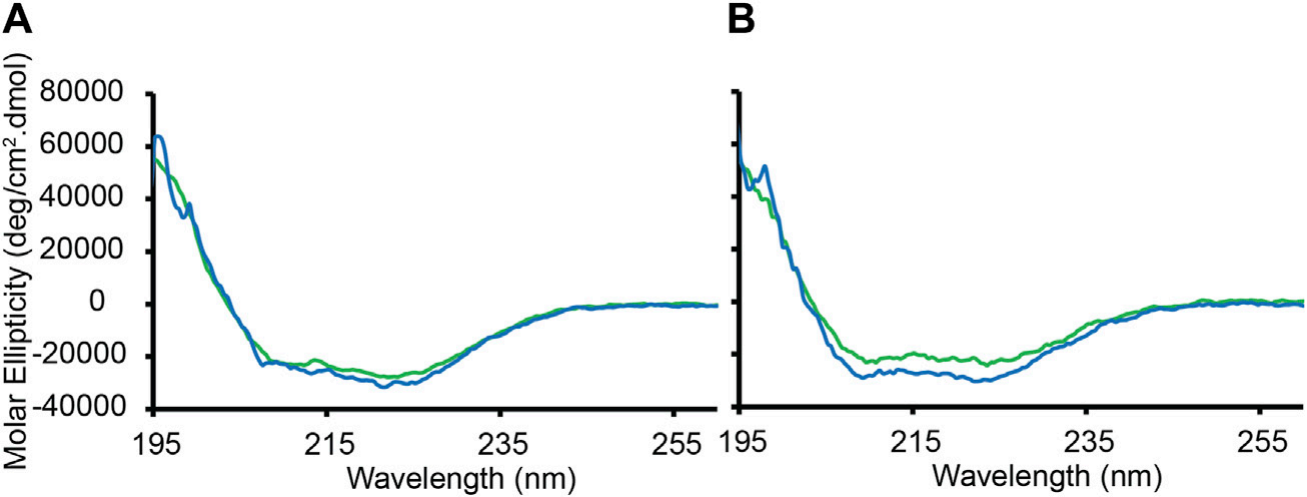
CD-monitored binding of P1 to large unilamellar vesicles containing L_o_ or L_o_/L_d_ phases. Large unilamellar L_o_/L_d_ and L_o_ vesiscles were prepared using DPPC/DPPE/POPC/POPE/Chol in ratios of 20:10:12:33:25 and 40:18:3:6:33, respectively. The vesicles prepared in phosphate buffer (5 mM potassium phosphate, pH 7.4) were extruded at a size of 0.1 µm and mixed with the peptide P1 at P/L = 1:120 (green) or 1:200 (blue). The peptide concentration was fixed to 20 μM. The molar ellipticity is displayed as a function of wavelength after subtracting the background signal from a lipid-only sample. **(A)** Binding to the L_o_/L_d_ mixture. **(B)** Binding to the L_o_ only. The data were recorded at 50°C, which is above T_low_ and T_mix_. Duplicates collected on two different batches of vesicles gave rise to similar data. Based on the molar ellipticity obtained at 222 nm when P/L = 1:200, the amounts of peptide bound to the L_o_/L_d_ and L_o_ are 98% and 92%, respectively.

Figure 4A shows the CD data obtained at 45 °C when P1 was added to the L_o_/L_d_ viral mimics at P/L = 1:200 and 1:120. Based on the ^2^H and ^1^H data presented in Figure 3 and Supplementary Figure S5, the L_o_ and L_d_ are miscible at this temperature. The CD bands detected at 208 and 222 nm demonstrate that P1 is α-helical, and therefore bound to the viral envelope mimics. The binding is stronger at lower P/L values, with 98% of the peptide bound at P/L = 1:200. Similar results are obtained with the L_o_ sample, with 92% binding detected at P/L = 1:200 (Figure 4B). Below 45°C, the CD data became noisier, preventing a similar characterization. However, NMR data (below) were collected at 30°C, showing that the peptide binds to the L_o_/L_d_ below T_low_.

### Permeabilization effects of P1 on L_o_/L_d_ and L_o_ viral envelope mimics

Next, we investigated whether P1 permeabilizes viral envelope mimics featuring the L_o_/L_d_ and L_o_. We previously used dye leakage assays to characterize the concentration-dependent permeabilization effects of P1 on various L_d_ membrane mixtures (Perrin et al., 2014; Hayden et al., 2015; Comert et al., 2019). It was found that P1 tends to be more membrane active when PC rather than PE headgroups are combined with anionic phosphatidylglycerol (PG), possibly because PE is more extensively hydrogen bonded and features stronger intrinsic negative curvature compared to PC (Murzyn et al., 2005). We also observed that P1 is active on zwitterionic membranes and Chol does not inhibit its membrane disruptive effects (Comert et al., 2019).

**FIGURE 5:**
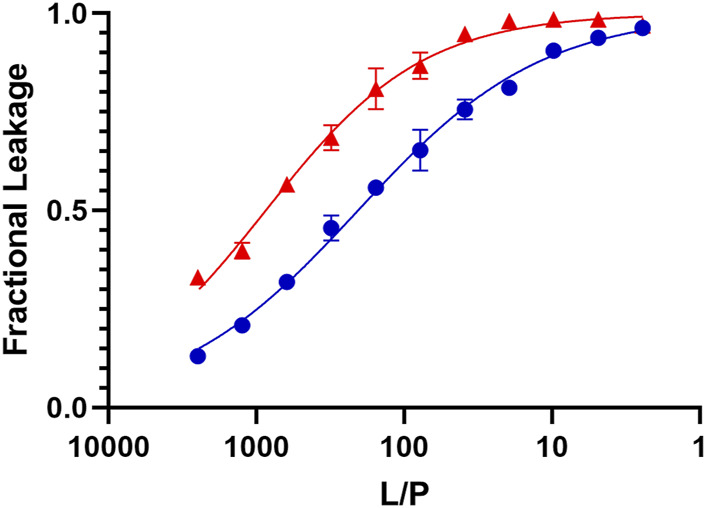
Dye leakage assays for L_o_ and L_o_/L_d_ phases viral envelope mimics exposed to P1. The fractional leakage is displayed for calcein dye leakage assays performed with P1 and large unilamellar vesicles containing the L_o_/L_d_ (red) and L_o_ (blue) phases. The normalized fluorescence intensity obtained after incubation at 45 °C is plotted against the lipid-to-peptide (L/P) ratio and fitted using an adaptation of the Hill equation. Each point represents the mean of triplicates and error bars are ± SD. The positive control (100% leakage) is obtained using 0.1% Triton X-100 (see Methods). These curves are used to extract the EC_50_, which represents the L/P values at which 50% leakage is observed. These values are higher for P1 acting on the L_o_/L_d_ (EC_50_ = 1769 ± 220) than L_o_ (EC_50_ = 463 ± 31). Similar but lower EC_50_ values were obtained at 30°C, which is below T_low_ (Supplementary Figure S6).

Figure 5 (red) shows the data obtained when we exposed L_o_/L_d_ LUVs containing trapped calcein to increasing peptide concentrations. Based on the fitted dose-response curves, the effective lipid-to-peptide molar ratio (L/P) needed to reach 50% lysis (EC_50_) is 1769 ± 220 at 45 °C. At 30°C, which is below T_low_, the EC_50_ became 865 ± 35, and thus remained low. These EC_50_ values indicate that the peptide is more effective on the L_o_/L_d_ than the previously studied 3:1 PC/PG (EC_50_ = 22), 1:1 PE/PG (EC_50_ = 10), PC (EC_50_ = 166), and 4:1 PC/Chol (EC_50_ = 130) lipid systems (the experimental error was indicated to be 20%) (Perrin et al., 2014; Hayden et al., 2015; Comert et al., 2019). We also tested P1 on the L_o_ phase alone. The data in Figure 5 (blue) show that the EC_50_ decreased compared to the L_o_/L_d_ mixture (EC_50_ = 463 ± 31 and 206 ± 7 at 45°C and 30°C, respectively) but the peptide remained highly permeabilizing. Remarkably, even at 30°C, P1 is much more effective on the L_o_/L_d_ than the zwitterionic 4:1 PC/Chol L_d_. As indicated in the introduction, the interface between domains can be a place of higher susceptibility for disruption by membrane-active agents (Schön et al., 2008; Blicher et al., 2009; Apellániz et al., 2011; Hao et al., 2018; Nasr et al., 2020; Kumar et al., 2022; Utterström et al., 2022).

### Cryo-EM studies of L_o_/L_d_ and L_o_ viral envelope mimics exposed to P1

**FIGURE 6:**
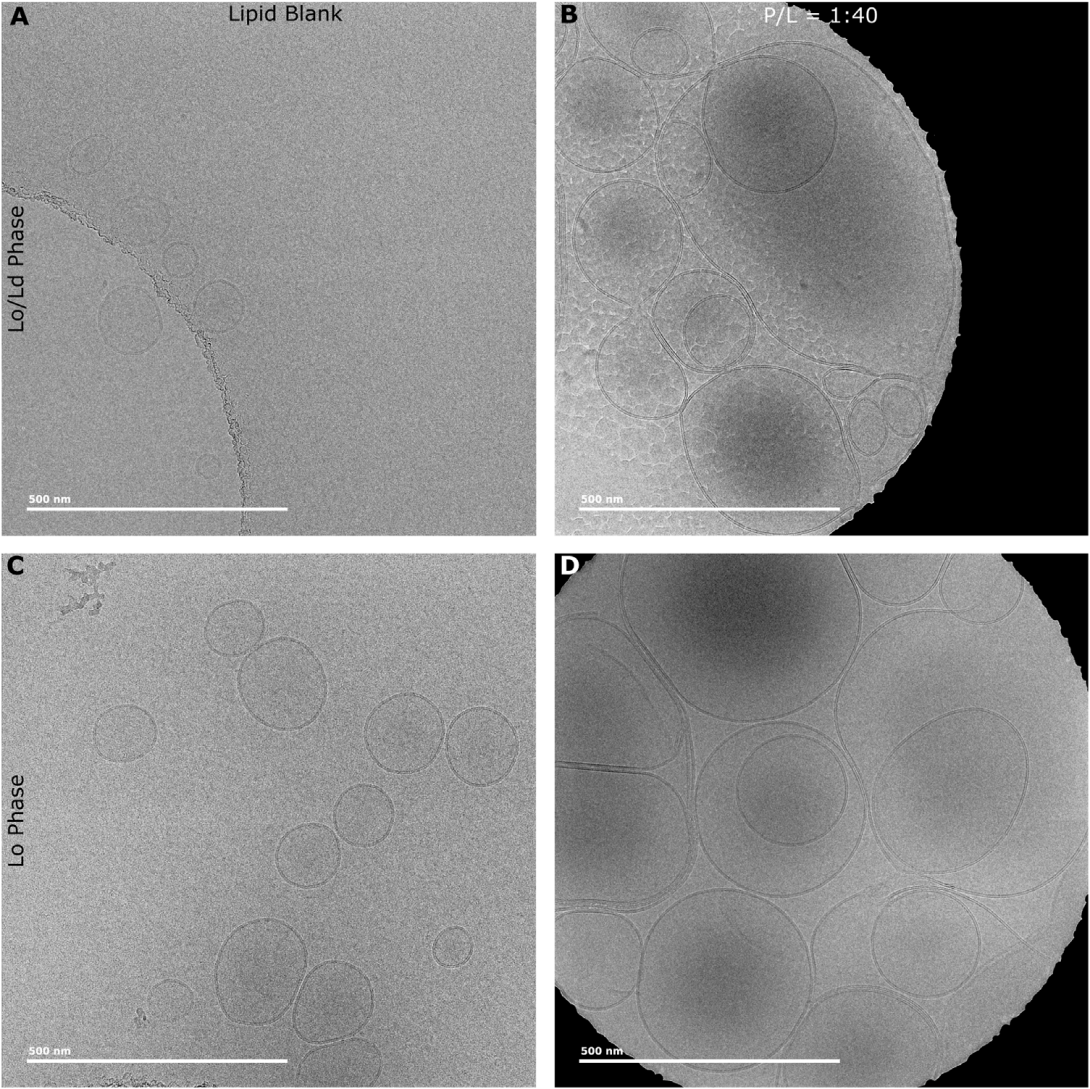
Cryo-EM micrographs of L_o_ and L_o_/L_d_ phases vesicles exposed to P1. LUVs (L_o_/L_d_ and L_o_) extruded at a size of 0.1 µm were exposed to P1 at P/L = 1:40. L_o_/L_d_ **(A)** and L_o_ **(C)** vesicles have normal unilamellar structure and size in the absence of piscidin. Upon adding the peptide at P/L = 1:40 to the L_o_/L_d_ **(B)** and L_o_ **(D)** vesicles, their size increases dramatically and multilamellar structures are observed. A ML process was developed to annotate the vesicles and extract the membrane thickness and curvature (See Methods). Supplementary Figure S8 shows an example of vesicles annotated every 4 nm as part of the ML analysis.

Previous studies with membrane-active HDPs, including P1, have showed that their binding to membranes induces thinning (Balla et al., 2004; Ouellet et al., 2006; Mihailescu et al., 2019). Knowing that P1 permeabilizes viral envelope mimics featuring a high Chol content, we employed Cryo-EM to investigate how thickness changes when these model membranes are exposed to the peptide. The L_o_ and L_o_/L_d_ vesicles used for Cryo-EM were first characterized by DLS (Supplementary Figure S7) to confirm size homogeneity. Figure 6 shows the Cryo-EM images obtained for such LUVs before and after exposure to P1. The data indicate that the membrane is dramatically rearranged when the peptide is present. From a visual inspection of the micrographs, the LUVs become much larger and adopt multilamellar structures, which indicates that aggregation and fusion occur upon peptide addition.

To quantify the changes in membrane thickness and vesicle size that P1 induces to LUVs, we developed a new automated program to annotate the micrographs and characterize their curvature over a distance of 4 nm. This protocol was inspired by previous work done by the Heberle lab where Cryo-EM data collected on LUVs were analyzed to yield bilayer thickness and relative amounts of L_o_ and L_d_ (Heberle et al., 2020). In that case, the analysis was done manually. Here, a ML process was leveraged to process large data sets and give direct information on vesicle size since curvature and vesicle size are inversely related. Major benefits of this approach include that it is fast, and since all of the vesicles are analyzed, bias towards prioritizing some vesicles over others is avoided. Supplementary Figure S8 shows examples of vesicles that were annotated by this method.

**FIGURE 7:**
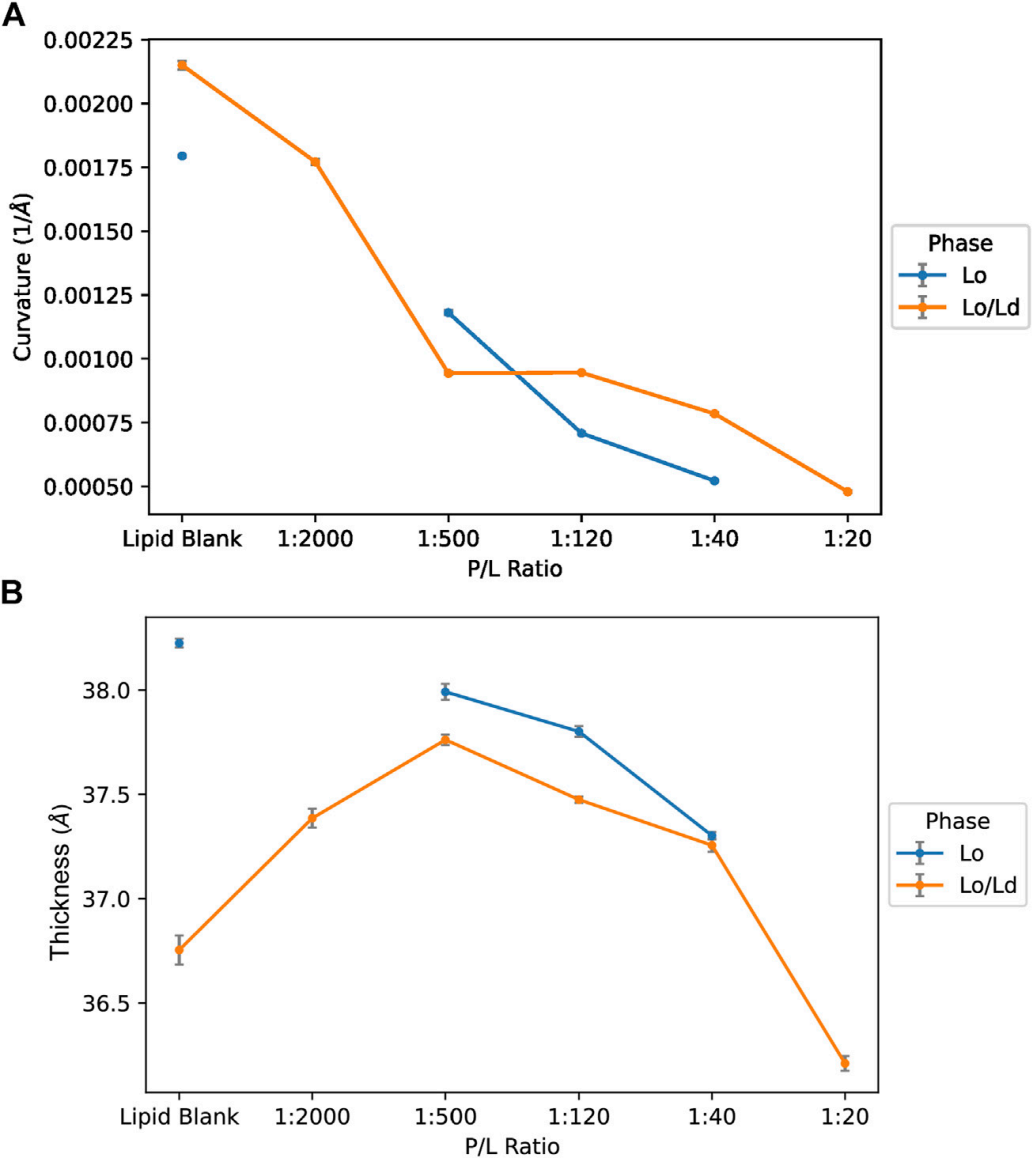
Curvature and thickness trends for vesicles containing L_o_ or L_o_/L_d_ phases as characterized by Cryo-EM data and ML. The data obtained by Cryo-EM were analyzed using an automated process to characterize the membrane curvature **(A)** and thickness **(B)** of the LUVs as a function of the P/L ratio (see Methods). Smaller vesicular curvature corresponds to larger vesicles. Smaller thicknesses indicate thinning of the bilayers. The error bar indicates ±3 SD.

As shown in Figure 7, a clear relationship exists between the curvature and P/L, with the curvature decreasing (i.e., the membrane becomes more flat as the vesicles become larger) when more peptide is added. This is observed whether the L_o_/L_d_ or L_o_ system is used. A trend towards reduced membrane thickness is also detected as more peptide is added. For the L_o_/L_d_ samples, the vesicles contain two types of domains, with the L_o_ phase being thicker due to higher Chol content. Since the analysis did not resolve the L_o_ from the L_d_, the trends represent how the thickness of the overall bilayer, i.e., the mixed L_o_ and L_d_ phases, responded to P1. When the L_o_ is used alone, thinning occurs but less than when the L_o_/L_d_ are mixed, indicating that the bilayer thinning effect of P1 occurs more readily when the peptide has the opportunity to interact with the L_d_ that it preferentially binds to (Comert et al., 2019). Overall, the statistical analysis indicates that the relationships associated with curvature and thickness are significant across the data sets: P1 decreases the curvature and thickness of the viral envelope mimics containing the ordered phase.

### Use of differential scanning calorimetry to investigate the ability of P1 to fuse and mix lipids

**FIGURE 8:**
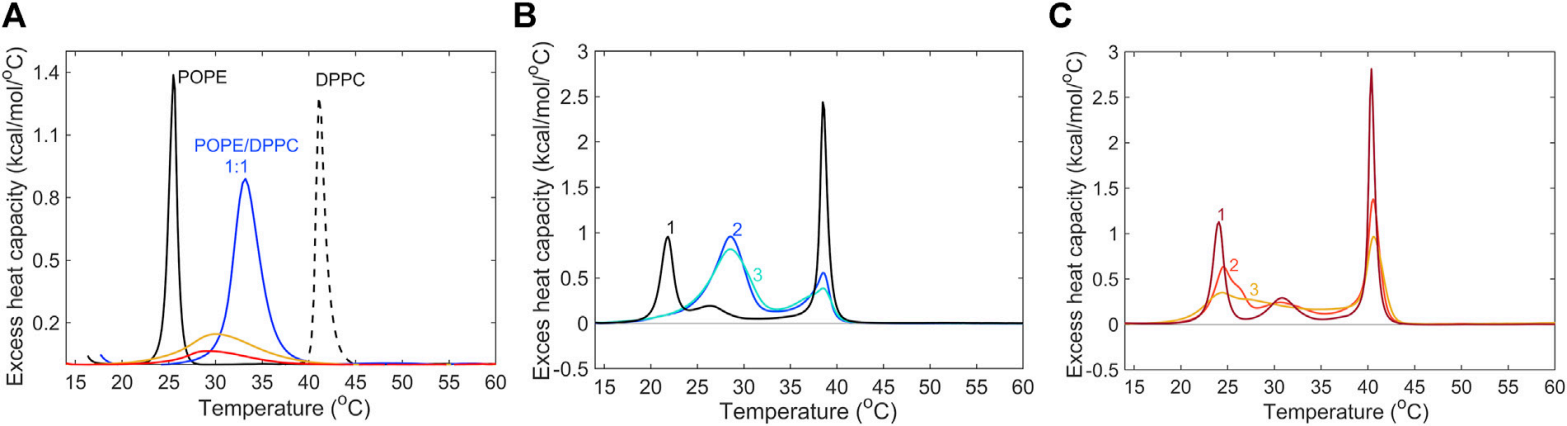
Differential scanning calorimetry data of model membranes exposed to P1. **(A)** The following conditions were investigated by DSC: gel-to-fluid transition curves for POPE (solid black), DPPC (dashed black), a homogeneous, equimolar mixture of POPE/DPPC (blue), a mixture of POPE/DPPC/Chol (1:1:0.5, molar) (yellow) and the same POPE/DPPC/Chol mixture in the presence of P1 peptide, at P/L = 1:100 (red). The samples were prepared at a target concentration of 3.5 mmol/L. The maxima of the melting transition temperatures (T_m_) were found at 25.4°C for POPE, 41.2 °C for DPPC, 32.8°C for POPE/DPPC (1:1), 30.1°C for POPE/DPPC/Chol, and 29.1°C for POPE/DPPC/Chol/P1. **(B)** Three consecutive heating scans (Lipsitch et al., 2022; Davis et al., 2023; Perumal et al., 2023) were performed on vesicles of POPE and DPPC in water, prepared separately (see Methods) and combined at a 1:1 ratio just before the measurements. The target total lipid concentration was 6.9 mmol/L. **(C)** DSC was performed on the same mixture of vesicles as in **(B)**, but the P1 peptide in water was added at P/L = 1:100 just before the measurements. See also Supplementary Figure S9 for full, raw heating, and cooling data.

Given the strong remodeling effect of piscidin on viral envelope mimics detected by Cryo-EM, we sought to characterize the ability of the peptide to mix lipids from vesicles varying in lipid composition. Investigations by DSC were aimed at observing the effect of P1 on the melting transitions and mixing behavior of lipids commonly found in raft mixtures such as POPE, DPPC and Chol, all of which are used to make our L_o_ and L_o_/L_d_ viral envelope mimics. Two types of experiments were performed. In the first type, POPE, DPPC and Chol were co-dissolved in chloroform to form a homogeneous lipid mixture that was then used to produce vesicles in water (Figure 8A). Taken separately, POPE and DPPC show the typical (gel-to-fluid) melting transitions at 25°C, and 41°C, respectively. However, when homogeneously mixed at a 1:1 molar ratio, they display a single transition peak at 30.8°C, broader than the two individual transition of the two lipids. The presence of Chol in proportion of 20% (molar fraction) broadened the transition of the mixture while shifting T_m_ to 29°C. Finally, addition of P1 to an aliquot of the same POPE/DPPC/Chol mixture in water further broadened the transition pointing to a strong lipid mixing and chain disordering effect due to P1. In a second approach, separate aliquots of POPE and DPPC vesicles were produced. We combined them with each other (Figure 8B) and with P1 in water (Figure 8C) just before the DSC measurements. We found that this type of investigation provides further insight into whether P1 has a differentiated effect on the two lipids. A few repeated scans were performed until the peak shapes stabilized (see also Supplementary Figure S9). As shown in Figure 8B, the POPE and DPPC melting peaks, while well separated at the beginning at the scans (T_m_ at ∼ 22°C and ∼39°C, respectively), eventually merged into a broad central peak (T_m_ ∼ 27°C), that was very similar to the transition peak for the pre-mixed POPE/DPPC (Figure 8A). However, when aliquots of the same POPE and DPPC vesicle suspensions in water were combined as above and P1 was added to this mixture immediately before the measurement, a much more dramatic mixing effect was observed (Figure 8C). By the third scan, the POPE peak completely disappears with no distinguishable mixed lipid peak. A remnant of the DPPC transition is still observed at 41°C. Overall, the data indicates that the P1 has an immediate effect on the melting and mixing of the two lipids, probably by promoting fusion of vesicles. Also, the POPE component appears to be more readily affected by P1. Not only does P1 cause “fluidization” and lipid mixing in membranes but it also displays affinities for certain components. In some of our previous studies, it was shown, by X-ray diffraction and microscopy in “raft” forming mixtures, that P1 has a preference to associate with fluid domains in membranes and/or causes Chol to segregate away from the P1-associated regions (Comert et al., 2019). Taken together, our DSC results indicate that P1 strongly affects the lipid landscape in mixed membranes such as those found in viruses. These changes in structure and organization are likely to directly affect the viral envelope lipids as well as affect viral protein distribution and function.

### Use of solid-state NMR to characterize the conformational arrangement of P1 bound to L_o_/L_d_ viral envelope mimics

We previously used oriented sample solid-state NMR to solve the structure of P1 bound to various lipid mixtures, including 3:1 PC/PG, 1:1 PE/PG, and 4:1 PC/Chol (Perrin et al., 2014; Comert et al., 2019). Not only does this method provide the structure of the peptide but it also characterizes the tilt and helix rotation in the membrane (Ketchem et al., 1997; Opella et al., 2001; Opella et al., 2002; Nevzorov et al., 2004; Opella and Marassi, 2004). Since P1 interacts with viral envelope mimics as shown by the data presented above, we made oriented samples for NMR structural analysis of P1.

**FIGURE 9:**
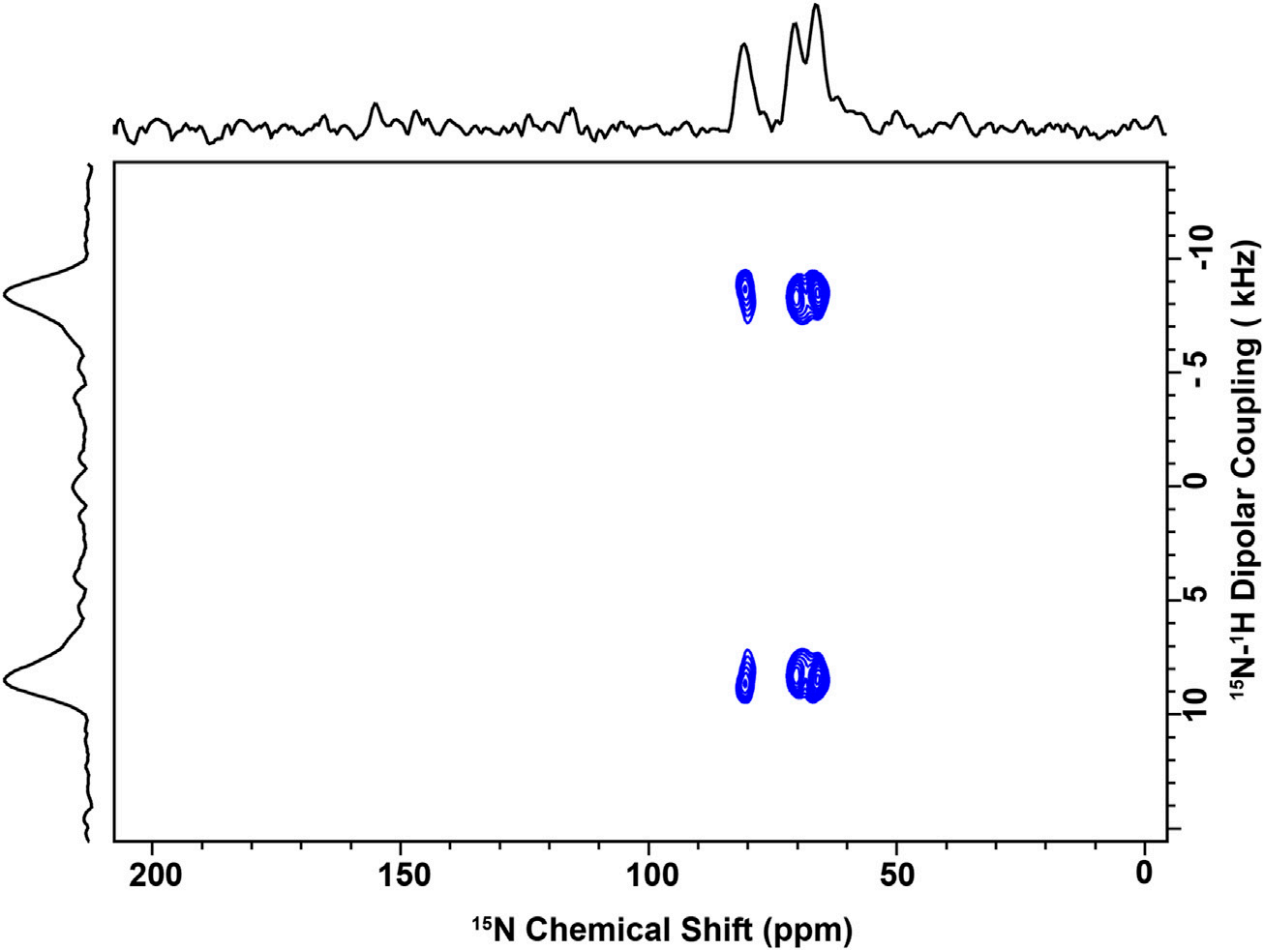
NMR spectrum of P1 bound to L_o_/L_d_ viral envelope mimics. 2D solid-state spectra of ^15^N-V_10_G_13_I_16_-P1 reconstituted in the L_o_/L_d_ phase. The sample was made at P/L = 1:100 and placed in the NMR probe with the bilayer normal parallel to B_0_. The data were recorded at ^1^H and ^15^N frequencies of 600 MHz and 60.8 MHz, respectively, with 2048 transients per slice of the 2D experiment. Each ^15^N label gives rise to a splitting in the indirect dimension, allowing for the quantification of the ^15^N-^1^H dipolar coupling between each amide ^15^N label and its attached proton. Based on previous studies of these three sites, the following assignments are made: V_10_: 80.5; G_13_: 70.1; and I_16_: 66.2 ppm. The spectrum was recorded at 30°C, which is below the miscibility point detected by NMR (See Figure 3; Supplementary Figure S5). Above T_mix_, a second set of splittings appeared in the indirect dimension suggesting that another peptide conformation was present (see Supplementary Figure S10).

Figure 9 shows the data obtained for ^15^N-labeled V_10_G_13_I_16_ P1 bound to the viral envelope mimics. Since the labeled positions are spread out along the backbone of the peptide, they report on the overall peptide structure, including G13 where the α-helix is kinked (Perrin et al., 2014). The data were collected at 30°C, which is below the T_low_ characterized by solid-state NMR. These spectra provide two orientational restraints per peptide plane: ^15^N chemical shifts (CSs) and ^15^N-^1^H dipolar couplings (DCs). Both of these sets of values (CS near 70–80 ppm and DCs around 8–10 kHz) are consistent with the peptide lying almost parallel to the membrane surface. Based on previous studies of these three ^15^N-labeled sites (Perrin et al., 2014; Comert et al., 2019), the signals were assigned as follows: V_10_: 80.5; G_13_: 70.1; and I_16_: 66.2 ppm. In comparison to the data obtained with the bacterial cell membrane mimic systems (PC/PG and PE/PG), the chemical shifts and DCs are comparable indicating similar tilt angles in the membrane, i.e., the peptide sits parallel to the membrane surface (Perrin et al., 2014; Comert et al., 2019). Hence, despite the high Chol content, P1 retains a strong ability to partition in these membranes. We also collected data at 45°C, which is above T_mix_, and noticed that a new set of resonances appeared in proximity to G_13_ and I_16_ while the V_10_ resonance became broader, and thus weaker (Supplementary Figure S10). This suggests that when the L_o_ and L_d_ are miscible based on the ^2^H data, the peptide adopts a minor conformational state, possibly reflecting that the peptide, which prefers interacting with the L_d_, is now able to partition significantly in the L_o_. Thus, its conformation or surrounding environment would be slightly different in the L_d_ than L_o_. This could result in different CSs and DCs given the sensitivity of these restraints.

## Discussion

The COVID-19 pandemic with its devastating death toll and societal impacts has underscored the crucial need to develop broad-spectrum therapeutics against (re)emerging viruses (Amanatidou et al., 2022; Lipsitch et al., 2022; Rahman et al., 2022; Zheng et al., 2022; Davis et al., 2023; Perumal et al., 2023). In this research, we feature a peptide-based approach to target viral membrane envelopes, given that their lipid composition and reparative abilities differentiate them from host cell membranes. Focusing on the membrane-mediated action of P1, a highly potent HDP with an aromatic-rich motif in its N-terminal region, we tested its antiviral activity on SARS-CoV-2 and investigated on a mechanistic level its disruptive effects on viral envelope mimics containing Chol. P1 demonstrates significant activity on both SARS-CoV-2 and HIV-1 (Wang et al., 2010; Wang, 2013), viruses with low and elevated cholesterol content, respectively (Brügger et al., 2006). We leveraged complimentary biophysical methods to establish structure-activity relationships in P1 acting on Chol-containing L_o_ and L_o_/L_d_ viral envelope mimics. A major finding from this work is that P1 is more active on phase-separated (L_o_/L_d_) membranes, indicating that P1 creates and exploits heterogeneity in mixed lipid bilayers by accumulating preferentially in L_d_ regions (Comert et al., 2019) and possibly, at regions featuring thickness mismatch-driven line tension at the boundaries between L_o_/L_d_ domains (Heberle et al., 2013). Next, we discuss the results and highlight important facets.

A novel feature of our work lies in the incorporation of ML tools to analyze large Cryo-EM data sets collected on lipid vesicles. Previously, Heberle and coauthors manually annotated vesicles as part of a study demonstrating the power of Cryo-EM to characterize the thickness of complex membranes such as L_o_/L_d_ mixtures (Heberle et al., 2020). Building on this, we developed a new methodology that automatically annotates individual vesicles, enabling the rapid tabulation of membrane thickness and curvature. This breakthrough has important ramifications given that vesicles are used in a growing number of fields such as membrane biophysics, medicinal chemistry, and drug delivery (Sheynis et al., 2003; Lo et al., 2015; Pattni et al., 2015; Hou et al., 2021; Liu et al., 2022; Utterström et al., 2022).

In recent years, peptide-based approaches have garnered interest in biomedical research and the pharmaceutical industry (Thundimadathil, 2012; Uhlig et al., 2014; Fosgerau and Hoffmann, 2015; Davenport et al., 2020; Varanko et al., 2020; Muttenthaler et al., 2021). This growth is bolstered by pivotal advances such as lower production cost, novel formulation methods, and the innovative incorporation of non-canonical amino acids that increase plasma half-life. In the context of fighting viruses, antiviral HDPs present several advantages, including the dual ability to decrease viral infectivity rates and exert anti-inflammatory effects on host cells. Among the few HDPs that have demonstrated antiviral action, P1 stands out due to its strong potency (EC_50_ = 5.4 μg/mL) against HIV-1 (Wang et al., 2010; Wang, 2013), a virus characterized by a Chol-rich envelope (Brügger et al., 2006).

In this study, we show that P1 is similarly active (EC_50_ ∼ 5 μg/mL) on SARS-CoV-2 and that its activity is partly due to direct action on the virions. Examples of synthetic antimicrobial peptides active on SARS-CoV-2 include MXB-4 and MXB-9 with respective EC_50_ of 20 and 7 μg/mL (Diamond et al., 2021), and thus less effective than Pl. DP7 is active on the SARS-CoV-2 protein pseudovirus at a potency (EC_50_ = 73.6 μg/mL) lower than that of P1, which was tested on the whole virus (Zhang et al., 2021). Our assays also show that P1 is more active than Brilacidin, a short HDP-mimetic that has gone through stage 2 clinical trials for its antimicrobial action. Previous reports indicated that P1 is particularly active on porcine epidemic diarrhea CoV (EC_50_ = 1 μg/mL) and pseudorabies (EC_50_ = 0.23 μg/mL) (Lei et al., 2018; Hu et al., 2019). Based on their egress pathways, these viruses are bound to have a low Chol content (Hogue et al., 2014; Schoeman and Fielding, 2019; Ghosh et al., 2020). Overall, these studies reveal that P1 is active on a broad range of viruses. Its activity on HIV-1 is particularly significant given that Chol increases the order and stability of membranes (Mihailescu et al., 2011). This led us to investigate on a molecular level how P1 achieves this uncommon effectiveness.

In earlier work that we performed, P1 was demonstrated to be active on Chol-containing zwitterionic membranes (Comert et al., 2019). More specifically, we found that P1 induces phase separation in homogeneous membranes, highlighting a novel mechanism of action where the peptide pushes away cholesterol and concentrates itself in the disordered region. Here, to characterize how P1 exerts its direct antiviral effects, we took an interdisciplinary approach. We employed antiviral and permeabilization activity assays to quantify the activity of P1 on SARS-CoV-2. The results demonstrate that its permeabilization effects have the following ranking: L_o_/L_d_ (20:10:12:33:25 DPPC/DPPE/POPC/POPE/Chol) > L_o_ (40:18:3:6:33 DPPC/DPPE/POPC/POPE/Chol) > L_d_ (4:1 POPC/Chol). Furthermore, P1 is active on the L_o_ alone, demonstrating its ability to interact with Chol-rich regions. Its stronger activity on phase-separated than homogenous bilayers supports a mechanism of disruption where enhanced activity is achieved by targeting the interface between the L_o_ and L_d_ phases. This phenomenon has been reported for several peptides involved in viral fusion (Schön et al., 2008; Blicher et al., 2009; Apellániz et al., 2011; Hao et al., 2018; Nasr et al., 2020; Kumar et al., 2022; Utterström et al., 2022). To the best of our knowledge, P1 could be the first HDP shown to interact with the L_o_ and target the L_o_/L_d_ interfaces.

To investigate the mechanism by which P1 disrupts L_o_ and L_o_/L_d_ membranes, CD, NMR, Cryo-EM, and DSC were combined. CD and ^15^N SS-NMR data document that the peptide adopts an α-helical structure that lies parallel to the surface of the viral envelope mimics. Cryo-EM and DSC reveal the strong lipid mixing effect of P1 on these membranes. The disordering effect of P1 is also demonstrated through the ^2^H SS-NMR experiments that establish the destabilization of the L_o_ domains in the L_o_/L_d_ mixture. Since P1 is able to interact with the L_o_, its inhibitory effect on demixing of the L_o_/L_d_ could be due to its inherent ability to partition in the region between the headgroups and hydrocarbon core, where it can induce disorder in the acyl chains of the lipids (Perrin et al., 2014; Hayden et al., 2015; Perrin et al., 2015; Mihailescu et al., 2019; Comert et al., 2021; Liu et al., 2023). Interestingly, P1 is more active on the L_o_ than L_d_ when each of them is used alone. As learned from earlier work with POPC/Chol bilayers, P1 can induce heterogeneity in homogenous membranes by pushing away the cholesterol (Comert et al., 2019), This provides a pathway to enhanced activity via the formation of Chol-depleted domains and defect-prone interfaces between domains. The N-terminal motif of P1, which is rich in aromatic residues, may promote its ability to initiate interactions with the L_o_ phase via π-stacking interactions (Epand et al., 2005; Apellániz et al., 2011; Zhou and Xu, 2012; Fantini and Barrantes, 2013; Paterson et al., 2017). Other nonpolar residues such as Ile and Val are also expected to play a role in mediating favorable interactions between the peptide and hydrophobic membrane. Another notable feature is that the fast bi-axial diffusion that apo P1 experiences about its α-helical axis and the bilayer normal enables the peptide to travel a distance that is greater than 30 μm per second (Chekmenev et al., 2010). The fast dynamics and compact arrangement of the α-helical peptide could translate into an enhanced capability to migrate to line defects and/or insert and disrupt the bilayer regions where it partitions.

Another aspect that could promote the disruption of viral envelopes by P1 is its preferential interaction with lipids that are important for viral fusion. As shown by the DSC data, the peptide prefers interacting with POPE over DPPC. Due to its inverted conical shape, the PE headgroup has a strong preference for the inverted hexagonal HII phase (Cullis and de Kruijff, 1979). Previous work that we performed showed that P1 inhibits PE from forming the HII phase, an effect that correlates with its ability to induce positive curvature strain (Perrin et al., 2015; Comert et al., 2019). As a result, it could hamper the formation of viral fusion intermediates since these feature negative spontaneous curvature.

Overall, P1 can leverage several effects to enhance its disruptive effects on viral envelopes that contain Chol. It can induce chain disordering, lipid mixing, and Chol-segregation. It exploits phase-separated membranes for enhanced permeabilization. Importantly, these effects can not only directly disintegrate viral envelopes but also indirectly disrupt the functions of key viral proteins embedded in the envelope. In particular, the disruption of lipids surrounding the glycosylated proteins (e.g., Spike proteins) involved in viral fusion could have deleterious effects on infectivity (Wolf et al., 2010; Miguel et al., 2013; Schoggins and Randall, 2013; Vigant et al., 2015; Yang et al., 2016). It would be insightful to investigate the role of these proteins in future mechanistic work on the peptide. Beyond acting on the viral membrane proteins, the peptide may be involved in changing the pH of endosomes, an effect reported for several antiviral peptides (Miller et al., 2011; Zhao et al., 2016). Further studies are needed to better understand the indirect effects of P1. Additional investigations could also be performed to compare P1 to other antiviral peptides, test its antiviral effect on other cell lines, and characterize its selectivity index. Examples of other HDPs with antiviral activity include caerin, indolicidin, LL-37, and maculatin (Wang, 2013; VanCompernolle et al., 2015; Ahmed et al., 2019; Hu et al., 2019; Ahmed et al., 2020).

In conclusion, this investigation unveils that P1, a peptide with an aromatic-rich N-terminal motif, is unique among HDPs by exhibiting promising antiviral properties against viruses such as HIV-1 and SARS-COV-2. It achieves stronger activity on HIV-1 and SARS-CoV-2 than other antimicrobial peptides. Our mechanistic studies on Chol-containing viral envelope mimics underscore several features important to explain its antiviral potency. First, P1 causes a melting of the gel phase of lipids by inserting with high affinity in the hydrocarbon core and inducing lipid mixing and acyl chain disordering. Second, P1 leverages heterogeneity in Chol-containing phase-separated membranes by preferentially interacting with the Chol-depleted region, thus exacerbating the existing line tension between regions and creating defects. Third, it induces positive curvature that counteracts the natural negative curvature of lipids such as PE. Together these local membrane effects could result in the direct disruption of viral envelopes as well as the indirect perturbation of surrounding membrane proteins. ML tools used in this study provided a novel way to rapidly annotate lipid vesicles and characterize their curvature and thickness. Overall, our combination of experimental and ML methods bodes well in the search for novel antiviral compounds that have broad spectrum properties and could help fight existing and (re)emerging viruses.

## Figures and Tables

**Table:** 

**APD**	Antimicrobial peptide database
**CD**	Circular dichroism
**Chol**	Cholesterol
**CI**	Confidence interval
**CoV**	Coronavirus
**COVID-19**	Coronavirus disease 2019
**Cryo-EM**	Cryogenic electron microscopy
**CS**	Chemical shift
**DMEM**	Dulbecco’s modified Eagle’s medium
**DPPC**	1,2-dipalmitoyl-sn-glycero-3-phosphocholine
**DPPE**	1,2-dipalmitoyl-sn-glycero-3-phosphoethanolamine
**HPLC**	High performance liquid chromatography
**DC**	Dipolar coupling
**DLS**	Dynamic light scattering
**DSC**	Differential scanning calorimetry
**EC50**	Concentration needed to reach 50% effectiveness
**ER**	Endoplasmic reticulum
**FBS**	Fetal bovine serum
**HIV-1**	Human immunodeficiency virus Type 1
**HDP**	Host defense peptide; hpi: hours post infection
**Ld**	Liquid disordered
**Lo**	Liquid ordered
**LUV**	Large unilamellar vesicle
**L/P**	Lipid-to-peptide molar ratio
**ML**	Machine learning
**MOI**	Multiplicity of infection
**MRSA**	*Staphylococcus aureus*
**P1**	Piscidin 1
**P3**	Piscidin 3
**P/L**	Peptide-to-lipid molar ratio
**PC**	Phosphatidylcholine
**PG**	Phosphatidylglycerol
**PI**	Phosphatidylinositol
**PS**	Phosphatidylserine
**POPC**	1-palmitoyl-2-oleoyl-sn-glycero-3-phosphocholine
**POPE**	1-palmitoyl-2-oleoyl-sn-glycero-3-phosphoethanolamine
**POPG**	1-palmitoyl-2-oleoyl-sn-glycero-3-phosphoglycerol
**SARS-CoV-2**	Severe acute respiratory syndrome coronavirus 2
***S. pneumoniae***	*Streptococcus pneumoniae*
**SS-NMR**	Solid-state NMR.

## Data Availability

The raw data supporting the conclusion of this article will be made available by the authors, without undue reservation.
