# Supplementary material for: Antiviral activity of the host defense peptide piscidin 1: investigating a membrane-mediated mode of action
Source: Front Chem. 2024 Jun 26;12:1379192. doi: 10.3389/fchem.2024.1379192 (PMC11233706; doi:10.3389/fchem.2024.1379192)
Supplement: Supplementary file 1 [file Presentation1.pdf]

Supplementary Information

Antiviral Activity of the Host Defense Peptide Piscidin 1: Investigating a Membrane-mediated Mode of Action

Tristan Bepler[1], Michael D. Barrera[2], Mary T. Rooney[3], Yawei Xiong[3], Huihui Kuang[1], Evan Goodell[3], Matthew Goodwin[4], Elizabeth Harbron[4], Riqiang Fu[5], Ella Mihailescu[6], Aarthi Narayanan*[7], and Myriam L. Cotten*[3,8]

[1]New York Structural Biology Center, New York, NY, United States

[2]School of Systems Biology, George Mason University, Manassas, VA, United States

[3]Department of Applied Science, William & Mary, Williamsburg, VA, United States

[4]Department of Chemistry, William & Mary, Williamsburg, VA, United States

[5]National High Magnetic Field Laboratory, Tallahassee, FL, United States

[6]Institute for Bioscience and Biotechnology Research, Rockville, MD, United States

[7]Department of Biology, George Mason University, Manassas, VA, United States

[8]Department of Biochemistry and Biophysics, Oregon State University, Corvallis, OR, United States

Corresponding Authors

*Myriam L. Cotten, E-mail: mcotten@wm.edu; *Aarthi Narayanan, fax: (518)-276-3405; E-mail: anaraya1@gmu.edu

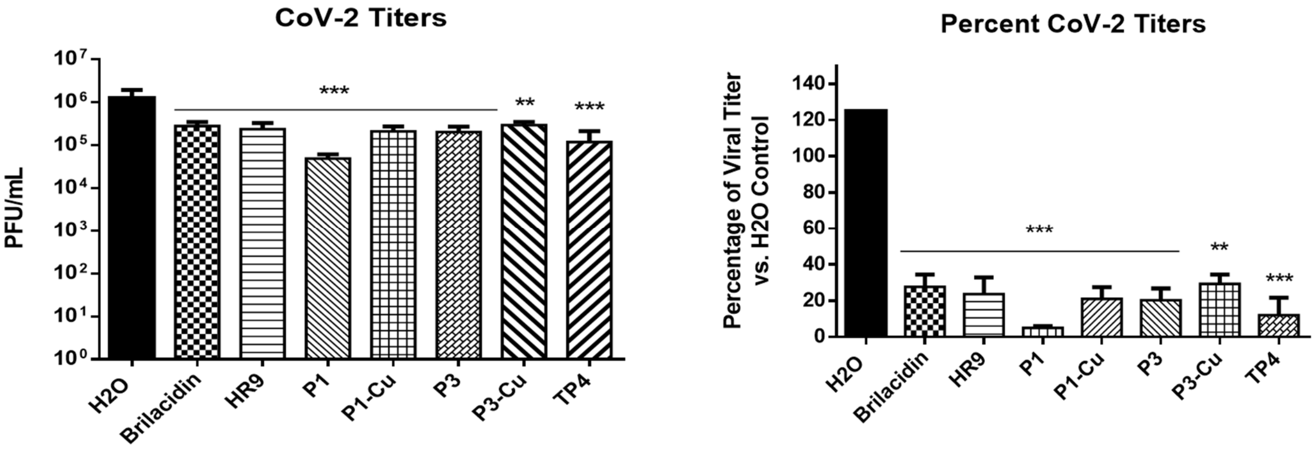

Figure S1: **Antiviral effects of Piscidin 1 and other host defense peptides on SARS-CoV-2.** Vero cells were employed to quantify the amount of SARS-CoV-2 infectious titer by plaque assay. Cells seeded at a density of 2×10^5 per well were pre-treated with the indicated peptides at a concentration of 10 µg/ml. Infection with SARS-CoV-2 was done at an MOI of 0.1. Data are reported as the average plaque forming units (Avg PFU/mL) over triplicates; $^{**}p > 0.01$ and $^{***}p>0.001$. Brilacidin, a peptoid previously shown to be active against SARS-CoV-2,[1] was used as a positive control. Plaque assay results are displayed as both plaque forming units per milliliter (PFU/mL) (left) and as percentage of virus titer versus the H_2O control (right). Among the peptides tested, P1 demonstrated the strongest inhibitory effect on SARS-CoV-2 followed by TP4. Under the conditions tested, it was more effective than Brilacidin.

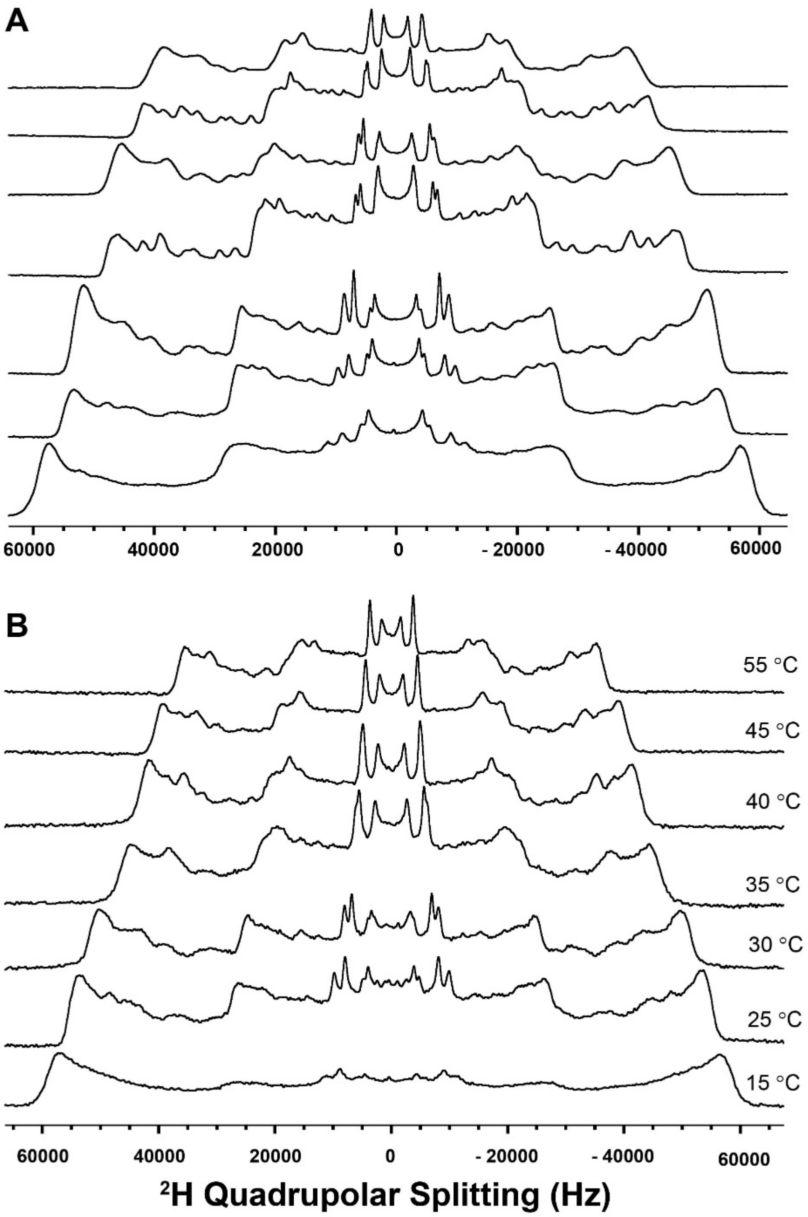

Figure S2: ^{2}H static solid-state NMR of L_o/L_d viral envelope mimics in the absence and presence of P1. Multilamellar vesicles prepared with 20:10:12:33:25 DPPC/DPPE/POPC/POPE/Chol were studied by ^{2}H static solid-state NMR at different temperatures in the absence (A) and presence of P1 at P/L = 1:100 (B). For ^{2}H detection of the phospholipid methyl region, deuterated DPPC (d62) was incorporated in samples mechanically aligned on glass plates. Quadrupolar splittings arising from deuterated methyl groups give rise to signals that are centered at 0 kHz. The full spectra giving rise to the data shown in Figure 3 are shown here.

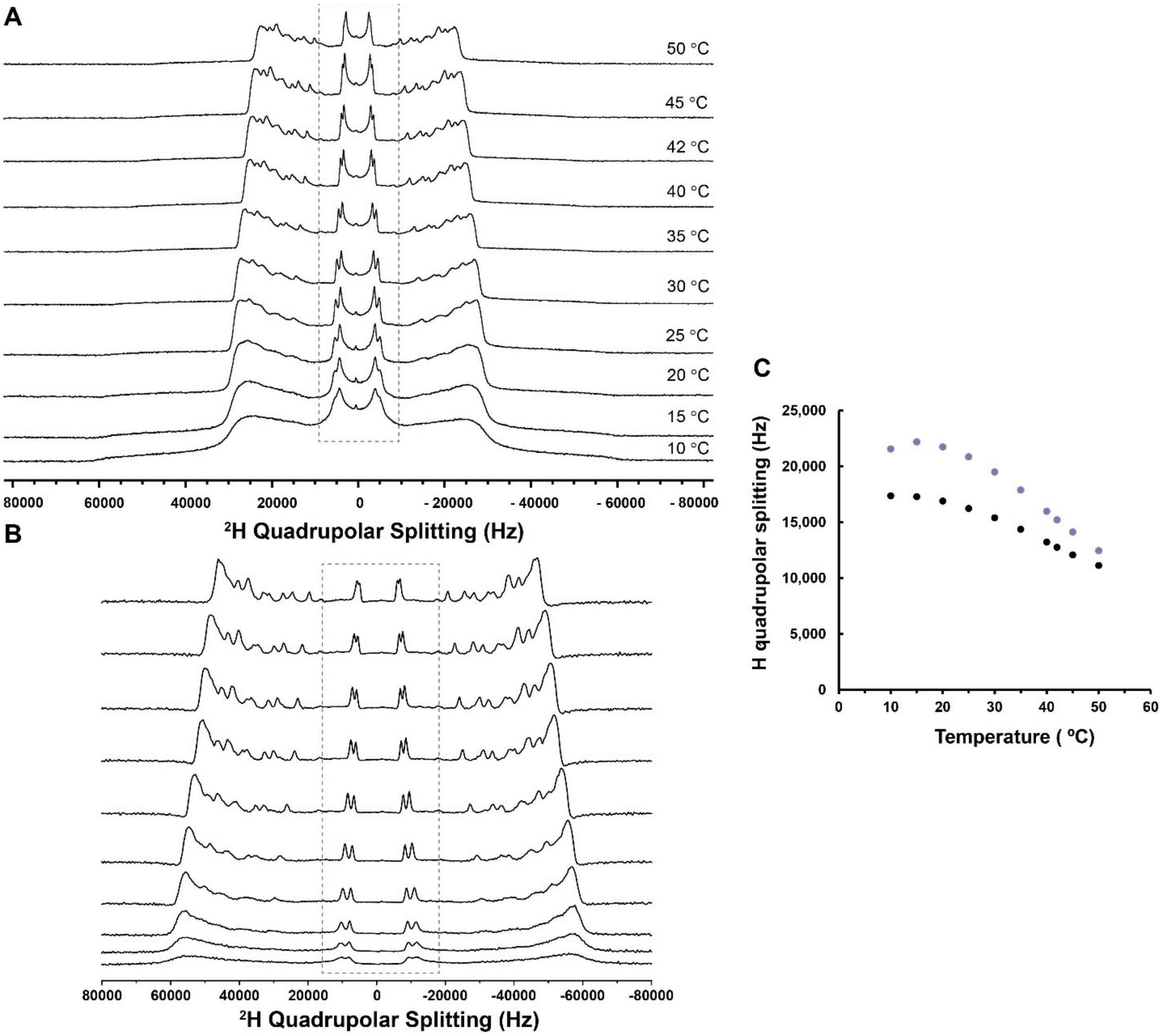

Figure S3: ^{2}H static solid-state NMR of L_o viral envelope mimics. Multilamellar vesicles prepared with 40:18:3:6:33 DPPC/DPPE/POPC/POPE/Chol were studied by ^{2}H static solid-state NMR at different temperatures. For ^{2}H detection of the phospholipid methyl region, deuterated DPPC (d62) was incorporated in the unoriented bilayers. A: Full spectra, with the methyl region boxed using dashed lines. These data were recorded on an Avance III console. B: Methyl region after de-Pakeing. The methyl region is also boxed using dashed lines. C: Plot of the methyl quadrupolar splitting versus temperature. Throughout the temperature range, two large splittings (> 5 kHz) are present, indicating that the L_o is present.

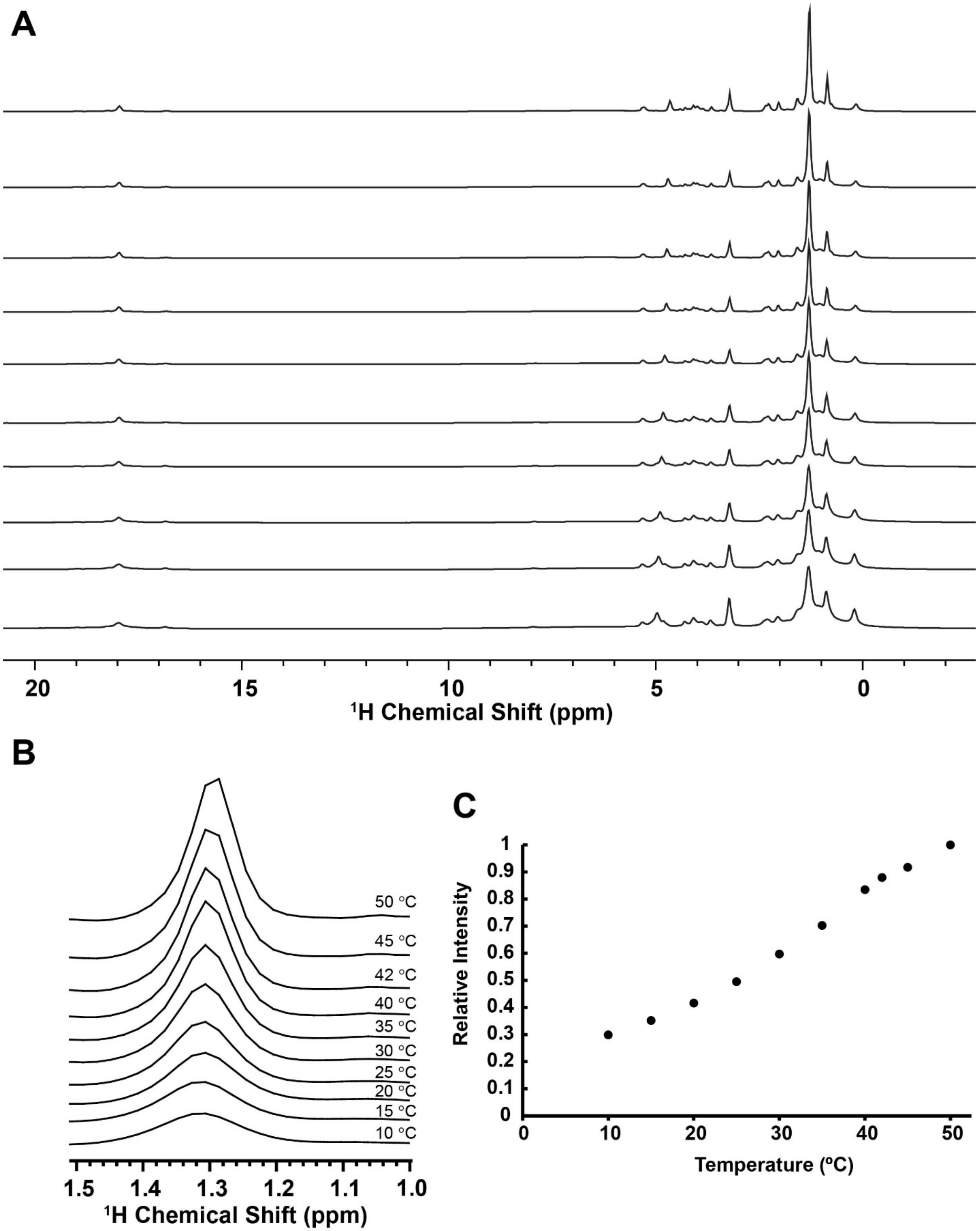

Figure S4: ^{1}H MAS solid-state NMR of L_o/L_d viral envelope mimics in the absence of P1. Multilamellar vesicles prepared with 20:10:12:33:25 DPPC/DPPE/POPC/POPE/Chol were studied by ^{1}H MAS NMR at different temperatures. A: Stacked ^{1}H MAS spectra as a function of

temperature. Intensities are not on the same scale. The samples were spun at 10 kHz, giving rise to sidebands. The sideband for the methylene region appears near 18 ppm on the 600 MHz instrument used for these experiments. B: The methylene region of the spectra shown in A are expanded and plotted on the same scale to show the drop in intensity. C: Plot of the methylene intensity versus temperature. Below T_{mix}, the sample is expected to experience a dramatic change in intensity, reaching only about 10-20% of its maximum height. Above T_{mix}, the intensity may still change but not as drastically.[2] The leveling off that happens at T_{mix} was not observed over the range of temperatures tested, thus $T_{mix} > 55$ ºC. From ^{2}H NMR, T_{low} was defined to be 45 ± 5 ºC (see main text).

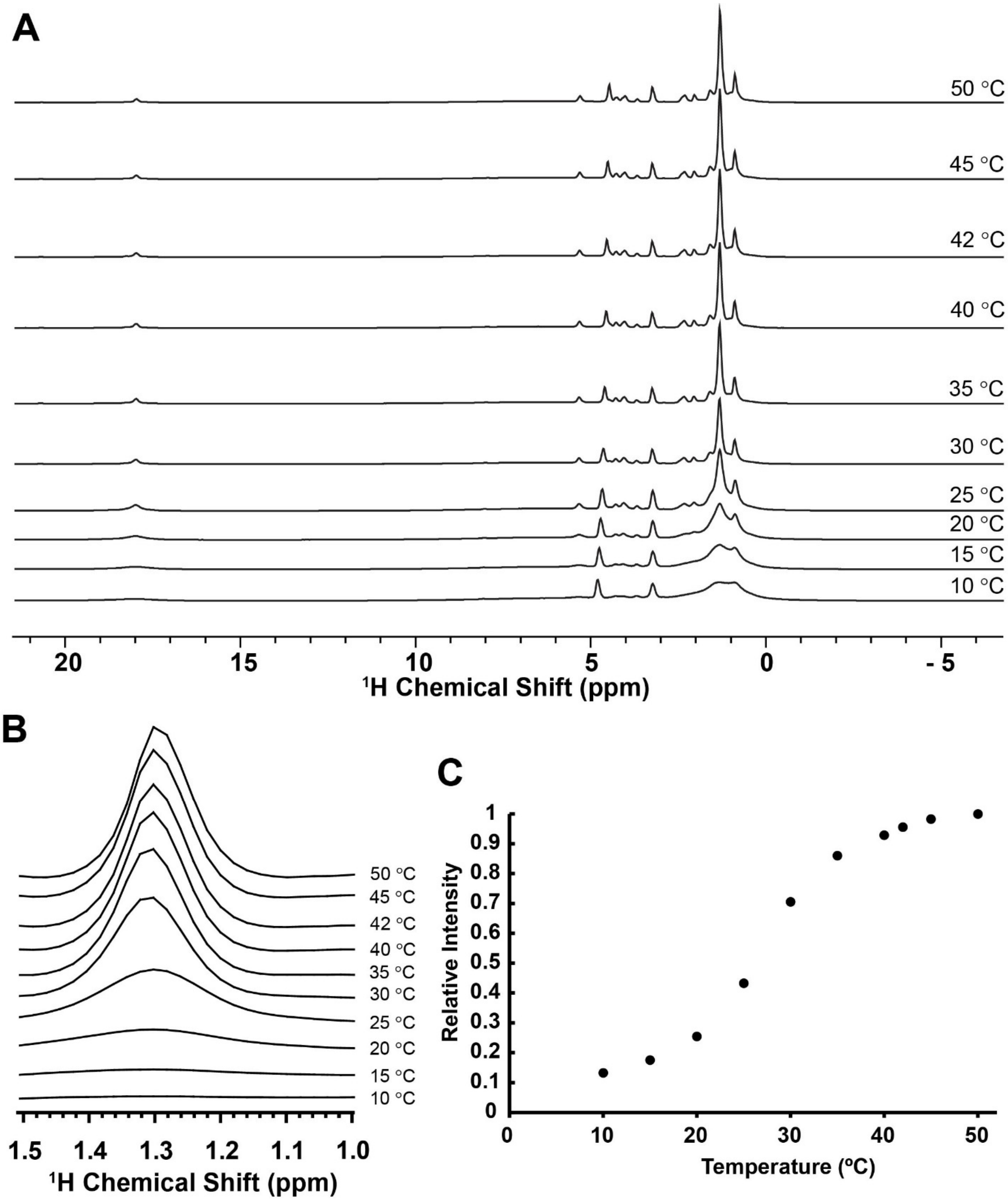

Figure S5: ^{1}H MAS solid-state NMR of L_o/L_d viral envelope mimics in the presence of P1. Multilamellar vesicles prepared with 20:10:12:33:25 DPPC/DPPE/POPC/POPE/Chol containing P1 at P/L = 1:100 were studied by ^{1}H MAS NMR at different temperatures. A: Stacked ^{1}H MAS spectra as a function of temperature. Intensities are not on the same scale. At the sample is cooled down, the linewidth increases. The samples were spun at 10 kHz, giving rise to

sidebands. The sideband for the methylene region appears near 18 ppm on the 600 MHz instrument used for these experiments. B: The methylene regions of the spectra shown in A are expanded and plotted on the same scale to show the drop in intensity. C: Plot of the methylene intensity versus temperature. Below T_{mix}, the sample starts experiencing a dramatic change in intensity while some leveling off occurs once T_{mix} is reached.[2] For this sample, the plateauing occurs near 40 ± 5 ºC, defining the value of T_{mix}. From ^{2}H NMR, T_{low} was defined to be 35 ± 5 ºC (see main text). This behavior of the sample differs from the lipid-only sample shown in Figure S4, confirming the ^{2}H NMR study showing that the peptide has a strong effect on the phase behavior of the lipids.

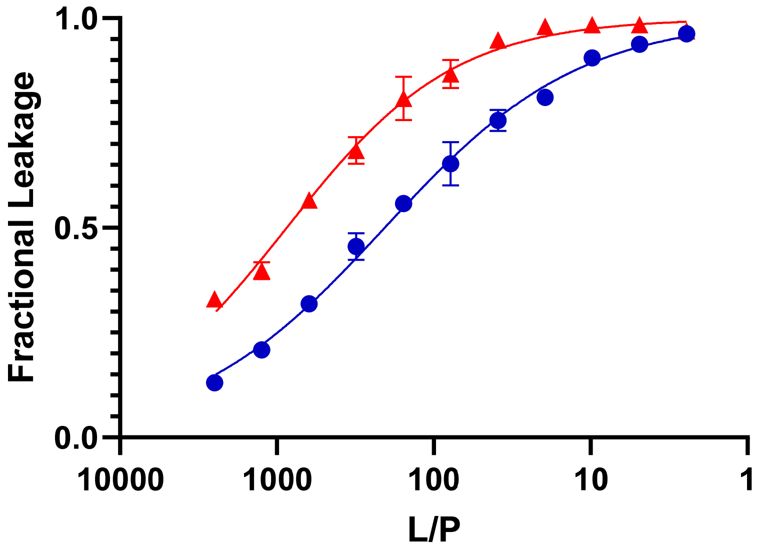

Figure S6: Dye leakage assays for viral envelope mimics exposed to P1. The fractional leakage is displayed for calcein dye leakage assays performed with P1 and large unilamellar vesicles (LUVs) containing the L_o/L_d (red) and L_o (blue) phases. The normalized fluorescence intensity obtained after incubation at 30 °C is plotted against lipid-to-peptide (L/P) ratio and fit using an adaptation of the Hill equation. Each point represents the mean of triplicates and error bars are +/- SD. The positive control is 0.1% Triton X-100 (see methods). These curves are used to extract the EC_{50}, which represents the L/P values at which 50% leakage is observed. These values are higher for P1 acting on the L_o/L_d (EC_{50} = 865 ± 35) than L_o (EC_{50} = 206 ± 7).

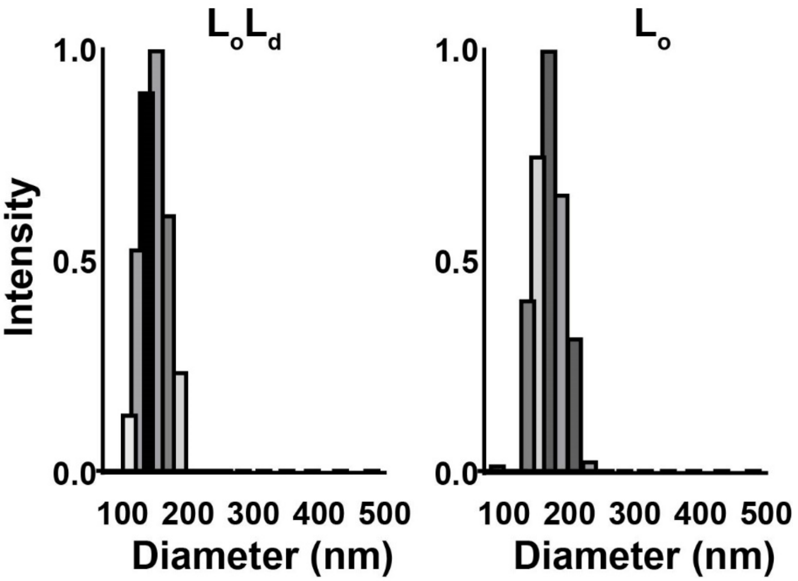

Figure S7: Dynamic Light Scattering data for characterization of LUV size. DLS was used to check the uniform size of the LUVs made by extrusion. Gaussian analysis done on three independent sets of vesicles yielded average sizes and standard deviations of 149 ± 10 and 165 ± 5 nm for the L_o/L_d and L_o, respectively. A representative data set is shown here. This is larger than the size of 100 nm used for the extrusion process, but Chol has an ordering effect and leads to thicker membranes.

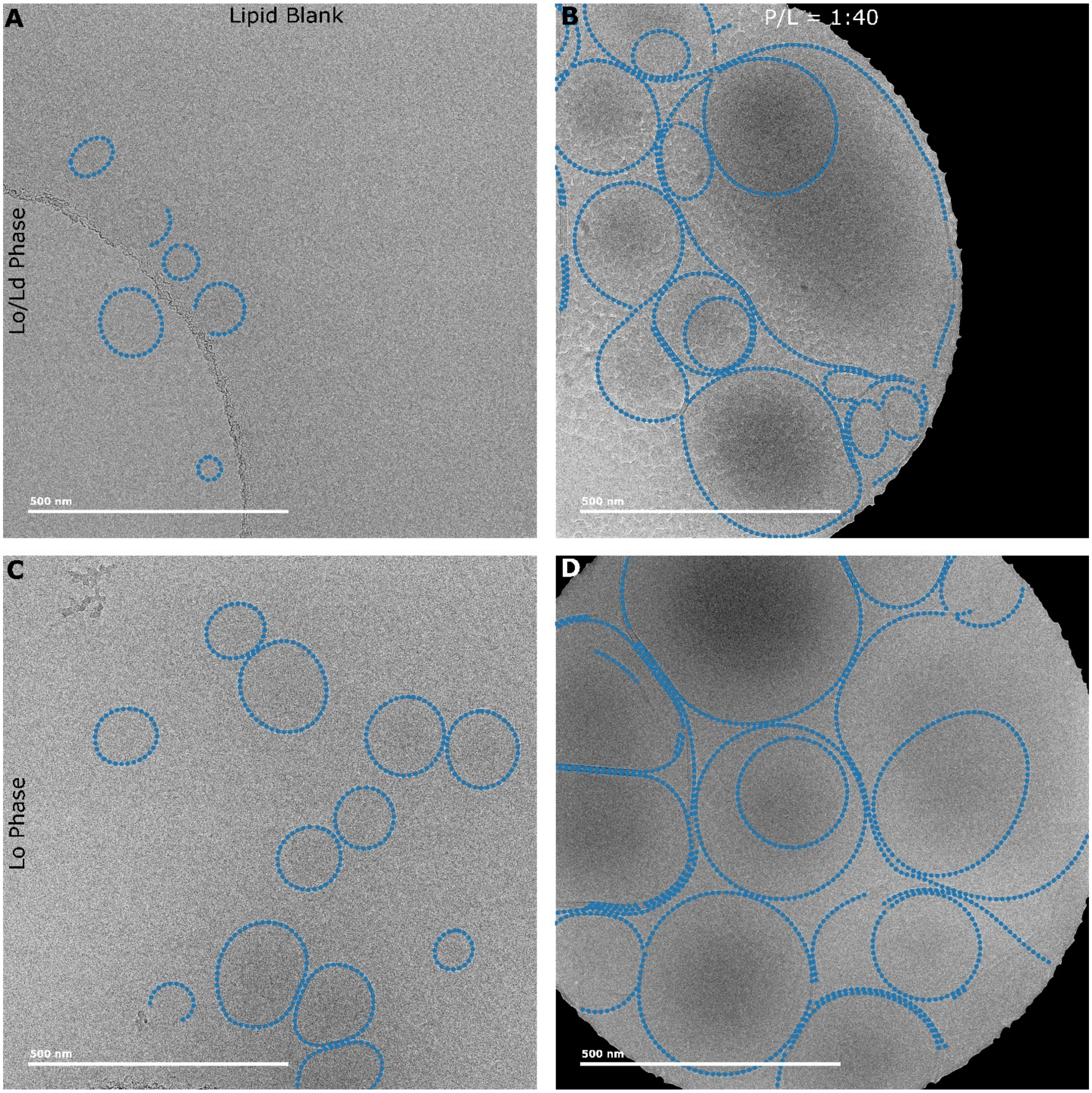

Figure S8: Cryo-EM micrographs annotated by machine-learning process. LUVs (L_o/L_d and L_o) extruded at a size of 0.1 μm were imaged alone or exposed to P1 at P/L = 1:40. This figure shows the annotation done by ML for the vesicles displayed in Figure 6 of the main text.

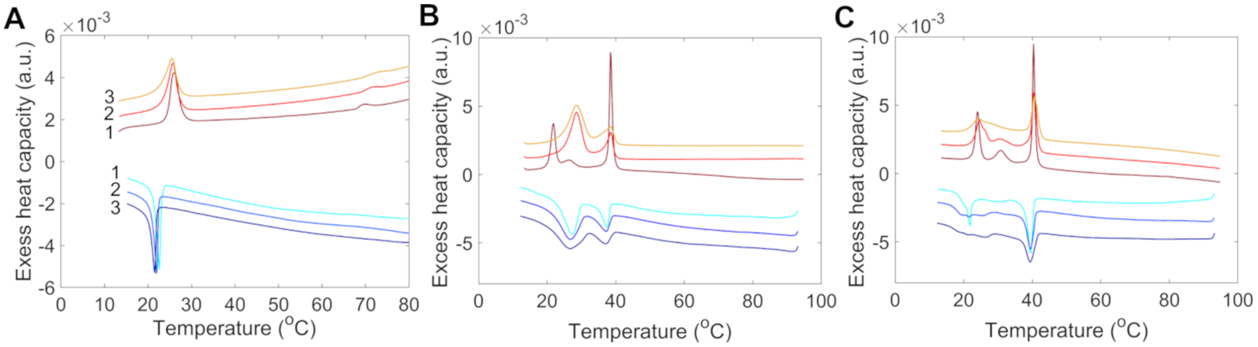

Figure S9: Raw DSC scans. (A) Raw scans of POPE prepared as a vesicle suspension in water at a concentration of 3.5 mmol/L. Three sets of consecutive heating (upper curves) and cooling scans (lower curves) were collected. POPE shows a strong gel-to-fluid transition at ~ 25 °C and a small lamellar to inverted hexagonal transition at ~ 71 ºC. (B) Three consecutive heating scans of vesicles of POPE and DPPC in water, prepared separately and combined at a 1:1 ratio just before measurement. The target total lipid concentration was 6.9 mmol/L. (C) Same mixture of vesicles as in (B) to which P1 peptide in water was added just before the measurement, for a P/L of 1:100.

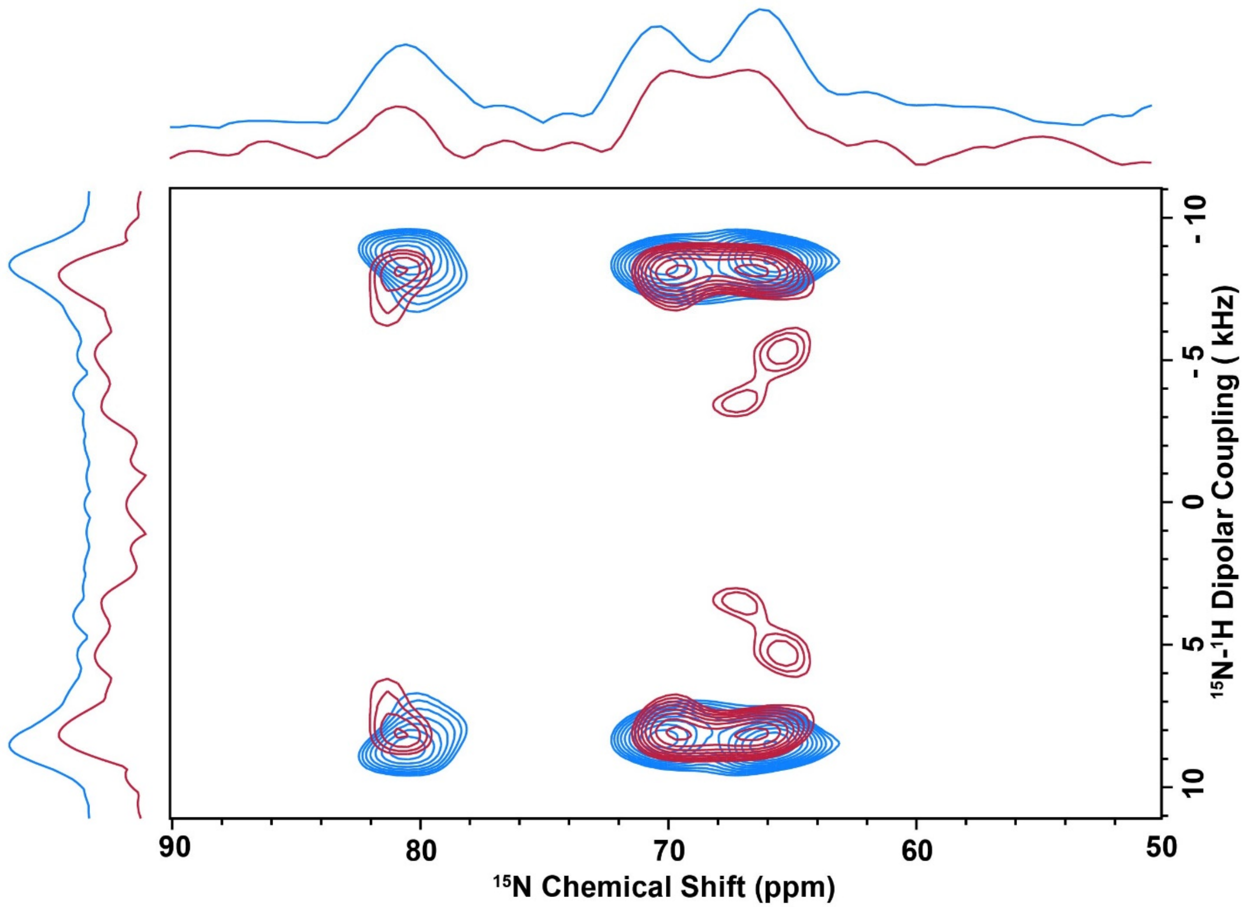

Figure S10: NMR spectra of P1 bound to L_o/L_d viral envelope mimics at two different temperatures. Data displayed in Figure 9 that were obtained at 30 °C (blue) are compared to a spectrum collected at 45 °C (red).

References

1. Bakovic, A.; Risner, K.; Bhalla, N.; Alem, F.; Chang, T. L.; Weston, W. K.; Harness, J. A.; Narayanan, A. Brilacidin Demonstrates Inhibition of SARS-CoV-2 in Cell Culture. *Viruses* **2021,** *13*.
2. Veatch, S. L.; Polozov, I. V.; Gawrisch, K.; Keller, S. L. Liquid domains in vesicles investigated by NMR and fluorescence microscopy. *Biophys J* **2004,** *86*, 2910-22.
